# Novel Bio-Inspired Physics-Based Learning and Evolutionary Guidance for Dynamic Multi-Objective Cold Chain Routings

**DOI:** 10.3390/biomimetics11060380

**Published:** 2026-06-01

**Authors:** Tongli He, Xiwen Yang, Wanzhen Huang, Fan Zhang, Guodong Li, Ze Niu, Jianhong Gan, Zhibin Li, Xun Deng, Tinghui Chen, Peiyang Wei, Shuai Li, Xiaoli Peng

**Affiliations:** 1 College of Applied Mathematics, Chengdu University of Information Technology, Chengdu 610225, China; htl@cuit.edu.cn; 2 College of Software Engineering, Chengdu University of Information Technology, Chengdu 610225, China; yangxiwen@casit.com.cn (X.Y.); hwc1968@outlook.com (W.H.); lizhibin111@outlook.com (Z.L.); dengxun22@mails.ucas.ac.cn (X.D.); chenth199208@outlook.com (T.C.); weipy@cuit.edu.cn (P.W.); 3 Faculty of Artificial Intelligence Education, Central China Normal University, Wuhan 430079, China; liguodong@ccnu.edu.cn; 4 Norinco Group Testing and Research Institute, Xi’an 710116, China; 18434360198@163.com; 5 Sichuan Key Laboratory of Software Automatic Generation and Intelligent Service, Chengdu University of Information Technology, Chengdu 610225, China; 6 Key Laboratory of Meteorological Software, China Meteorological Administration, Chengdu 610225, China; 7 Dazhou Key Laboratory of Government Data Security, Sichuan University of Arts and Science, Dazhou 635000, China; 8 Faculty of Information Technology and Electrical Engineering, University of Oulu, Pentti Kaiteran Katu 1, 90570 Oulu, Finland; shuai.li@oulu.fi; 9 VTT-Technical Research Centre of Finland, Kaitovayla 1, 90590 Oulu, Finland

**Keywords:** agricultural cold chain logistics, multi-objective optimization, dynamic route optimization, deep reinforcement learning, hybrid algorithm, H-MODRL

## Abstract

Agricultural cold chain logistics is characterized by inherent challenges—product perishability, high carbon emissions, and stringent time windows—which are further exacerbated by dynamic disruptions. Existing methods suffer from slow adaptability, unstable multi-objective convergence, and severe cold-start issues. This work falls within the broad scope of biomimetics—the science of emulating nature’s time-tested strategies to solve complex engineering problems—and bio-inspired data-driven methods and their applications in engineering control, optimization, and artificial intelligence. The proposed H-MODRL framework embodies core biomimetic principles: the Genetic Algorithm (GA) mimics Darwinian natural selection and genetic inheritance, the Sparrow Search Algorithm (SSA) abstracts the cooperative foraging and anti-predation behaviors of sparrow populations in nature, and the Arrhenius-based freshness-decay model captures the biochemical kinetics governing perishable biological products. By synergistically integrating these biological evolution principles, swarm intelligence, and deep learning, the framework tackles real-world logistics complexity in a manner directly inspired by living systems. This study presents a well-organized hybrid optimization framework (H-MODRL) that couples a three-stage hybrid evolutionary mechanism, synergistically integrating heuristic warm-start, evolutionary policy guidance, and deep reinforcement learning decision-making. First, an improved genetic algorithm combined with the earliest deadline first strategy constructs a feasible initial population satisfying hard time-window constraints. Second, a large neighborhood search-enhanced chaotic sparrow search algorithm builds a high-quality elite guidance set for policy learning. Third, a physics-based multi-objective proximal policy optimization model embedded with Arrhenius equation-derived freshness-decay kinetics performs online decision-making. Experiments demonstrate that pre-computed all-pairs shortest paths and an O(1) hash-based dynamic-disruption indexing mechanism support fast online replanning. On heterogeneous simulated terrains based on real Chinese geospatial data, H-MODRL outperforms state-of-the-art algorithms across four objectives—logistics cost, carbon emissions, terminal freshness, and delivery time—while exhibiting compact, low-variance performance distributions, thereby validating its engineering robustness and practical value in complex agricultural cold chain environments.

## 1. Introduction

In this paper, we propose a Hybrid Multi-Objective Deep Reinforcement Learning (H-MODRL) framework that synergizes physics-based modeling with a three-stage hybrid evolutionary mechanism to achieve efficient and robust dynamic routing in agricultural cold chain logistics under multiple conflicting objectives. Industrial cold chain logistics is a critical bridge between agricultural production and consumption, preserving food safety and product value [1,2]. As consumption patterns evolve, demand for high-value perishable agricultural products has surged [3,4,5]. Yet losses remain high because these products are intrinsically perishable, highly time-sensitive, and sourced from geographically dispersed producers [6]. At the same time, cold chain logistics is energy-intensive: refrigeration systems and transport fleets contribute substantial emissions, creating a major barrier to “carbon peak” and “carbon neutrality” targets in the transport sector [7,8]. In the context of consumption upgrading, new business models such as fresh e-commerce have expanded rapidly, and expectations for quality, speed, and low-carbon delivery continue to rise [9,10]. This makes it essential to optimize cost, timeliness, and carbon footprint without compromising freshness, thereby advancing both economic returns and environmental sustainability in smart agriculture and green logistics [11]. In line with the thematic focus on bio-inspired data-driven methods and their applications in engineering control, optimization, and AI, the proposed H-MODRL framework integrates biologically inspired evolutionary mechanisms with data-driven learning to address dynamic multi-objective cold chain routing, demonstrating how such hybrid paradigms can effectively handle real-world logistics complexities under uncertainty.

From a methodological perspective, the core algorithms employed in H-MODRL draw on biomimetic principles: the Genetic Algorithm (GA) abstracts Darwinian natural selection, the Sparrow Search Algorithm (SSA) mimics cooperative foraging and anti-predation behaviors of sparrow populations, and the Arrhenius-based freshness-decay model captures the biochemical kinetics governing perishable biological products. These bio-inspired components are synergistically integrated with deep reinforcement learning to address dynamic multi-objective cold chain routing in a manner informed by living systems.

The three-stage pipeline mirrors a hierarchical optimization strategy: Stage 1 (GA-EDF) constructs a feasible initial population to mitigate the cold-start problem commonly observed in DRL; Stage 2 (C-SSA-LNS) refines solutions through global exploration with chaotic mapping and large neighborhood search to build a diverse elite guidance set covering key regions of the Pareto front; and Stage 3 (MO-PPO) performs online adaptive decision-making in response to dynamic disruptions, leveraging the elite guidance set as informative priors. This staged design cleanly separates expensive offline training from lightweight online inference, enabling real-time responsiveness that pure heuristic methods cannot achieve while maintaining the solution quality characteristic of evolutionary search.

Research on agricultural cold chain logistics route planning (ACCL-RP) [12,13] has largely progressed along three directions: mathematical programming [14], metaheuristics [15], and deep reinforcement learning [16]. Early studies used exact methods such as branch-and-bound for small, static instances, but their scalability is limited. Metaheuristics, including genetic algorithms [17], ant colony optimization [18], and multi-objective evolutionary methods [19], then became dominant. For example, Wang et al. proposed an improved multi-objective genetic algorithm-based particle swarm optimization (MOGAPSO) algorithm to minimize distribution costs (including freshness-loss, refrigeration, and delay-penalty costs) and maximize customer time satisfaction in fresh-product distribution, incorporating a dynamic insertion algorithm for real-time orders, as validated through a case study in Shenzhen [20]. More recently, deep reinforcement learning has introduced an end-to-end decision paradigm with fast inference. Kong et al. [21] proposed a constrained hybrid pointer network that integrates graph neural network embedding and an attention decoder for collaborative multi-drone delivery optimization, demonstrating the potential of DRL in complex last-mile logistics. Liu et al. [22] provided a comprehensive review of reinforcement learning applications to the vehicle routing problem, covering methodological advances (e.g., Markov decision process formulations, integration with heuristics) and outlining future research directions, thereby underscoring the growing role of DRL in real-time dispatching systems.

However, translating these methods to real-world agricultural cold chain settings that are dynamic and constraint-heavy remains challenging. First, when jointly optimizing total cost, time-window violations, carbon emissions, and cargo freshness, existing DRL models often struggle to converge stably to high-quality Pareto fronts, and they handle physical constraints, such as temperature-control thresholds and hard time windows, only weakly [23]. Second, disruptions such as traffic congestion, vehicle failures, and urgent order insertions typically trigger expensive recomputation in conventional optimizers, delaying responses beyond real-time requirements [24]. Third, in large-scale heterogeneous road networks, DRL agents face vast exploration spaces and sparse rewards, making cold starts common and training inefficient [25].

To address these issues, we propose a well-organized hybrid optimization framework, termed Hybrid Multi-Objective Deep Reinforcement Learning (H-MODRL), that integrates three complementary paradigms within a unified physics-based framework: heuristic warm-start (GA-EDF), evolutionary policy guidance (C-SSA-LNS), and deep reinforcement learning decision-making (MO-PPO). Rather than proposing a fundamentally new DRL architecture, the contribution lies in the synergistic coupling of established techniques—each addressing limitations that the prior stage cannot resolve alone—into a coherent three-stage pipeline tailored for dynamic agricultural cold chain routing. Each stage addresses specific limitations that prior stages cannot resolve alone, forming a closed-loop feedback pipeline. Our contributions are threefold:

1. **Three-stage hybrid evolutionary framework.** We develop a complete pipeline spanning initialization, guidance, and decision-making. In Stage 1, an improved Genetic Algorithm (GA) combined with an Earliest Deadline First (EDF) strategy [26] rapidly generates feasible initial solutions under hard time-window constraints, mitigating the cold-start problem in DRL. In Stage 2, a Chaotic Sparrow Search Algorithm (C-SSA) [27] based on chaotic mapping [28] and a Large Neighborhood Search (LNS) operator [29] performs global exploration to construct a high-quality Elite Guiding Set, providing informative priors for the policy network. In Stage 3, we design a physics-based Multi-Objective Proximal Policy Optimization (MO-PPO) [30,31] model that integrates a mask mechanism and a reward function embedded with Arrhenius freshness-decay kinetics for accurate multi-objective decisions. The mask design is inspired by the Segment Anything Model (SAM) architecture and semi-supervised teacher–student schemes used by Gan et al. in medical image segmentation [32].
2. **Physics-based dynamics with fast online response capability.** We build a high-fidelity environment that explicitly integrates Arrhenius-based freshness-decay dynamics and a full life-cycle carbon emission model, improving realism for ACCL-RP [33]. All-Pairs Shortest Paths (APSP) precomputation [34] and an O(1) hash-based disruption query mechanism reduce event lookup and state update delays to the millisecond level during online operation. The complete online decision pipeline comprises three stages: O(1) disruption event query via hash indexing, fast state update via APSP matrix lookup, and PPO policy network forward inference.
3. **Full-spectrum multi-objective evaluation.** Using heterogeneous simulation scenarios built from real Chinese geospatial data, we establish a four-dimensional evaluation protocol covering total cost, time-window penalty, carbon footprint, and freshness loss. Experiments show that H-MODRL delivers markedly better overall performance under dynamic disruptions than NSGA-III [35], ALNS [36], and mainstream DRL baselines [37].

## 2. Problem Definition and Physics-Based Modeling

This section formulates agricultural cold chain logistics route planning under dynamic disruptions (ACCL-RP) as a multi-objective optimization problem on a dynamic graph. We then develop a freshness-decay model and a full life-cycle carbon emission model grounded in chemical reaction kinetics, and finally construct a mathematical program with four conflicting objectives. Real-time forecasting of dynamic disruptions can draw on the dynamic spatio-temporal graph convolutional network proposed by Gan et al. [38] to capture spatio-temporal dependencies among road-network nodes and enable millisecond-level traffic-state sensing. Building on this, a multi-timescale state prediction model for cold chain logistics can be constructed by combining the bidirectional temporal convolutional network and attention mechanism proposed by Gan et al. [39].

### 2.1. Dynamic Graph Structure and Disturbance Model

We model the agricultural cold chain logistics network as a time-dependent directed graph G(t) = (V, E(t)) [40]. The node set is V = {0}∪C, where node 0 denotes the depot, and C = {1, 2, …, N} denotes N customer nodes such as farms or supermarkets. The edge set E(t) = {(i,j)|i,j ∈ V, i ≠ j} varies over time to reflect evolving road conditions. Road-network dynamics are driven by a set of stochastic disruption events Ω. Each disruption is defined as δ_k_ = (I_ij_,τ_start_,τ_end_,α,β)∈ Ω, where I_ij_ identifies the affected road segment, [τ_start_,τ_end_] is the active time window, α is the speed attenuation factor, and β is the temperature-control failure factor. Under these disruptions, the travel time on segment (i,j) becomes a piecewise function of the entry time t:

(1) $$T_{ij}(t)=\frac{d_{ij}}{v_{ij}\prod_{\delta_{k}\in \Omega }\left(1-I(t,\delta_{k})(1-\alpha_{k})\right)}$$

where d_ij_ is the segment distance, v_ij_ is the nominal design speed, and I(t,δ_k_) is an indicator function that equals 1 if t falls within the time window of δ_k_ and 0 otherwise. To support millisecond-level online queries, the implementation uses a hash-based O(1) dynamic indexing mechanism to store and retrieve Ω.

### 2.2. Freshness-Decay Kinetics of Agricultural Products

Freshness loss in high-value perishables is an irreversible thermodynamic process [41]. Instead of approximating loss by a linear time discount, we model freshness decay using the Arrhenius equation and first-order reaction kinetics, yielding a coupled temperature–time formulation. Let the product p have initial freshness θ_0_ = 1.0. The remaining freshness at time t is defined as

(2) $$\theta_{p}(t)=\theta_{0}\exp(-\int_{0}^{t}\lambda_{p}\eta (temp(\tau ))d\tau )$$

where λ_p_ is the intrinsic decay coefficient of product p at a reference temperature, and η(temp(τ)) is the temperature-sensitivity factor. When temperature-control failure occurs, represented by a disruption with β > 1, the cargo temperature increases, η rises sharply, and freshness loss accelerates. The cargo loss cost at customer i with demand q_i_ upon arrival at time t_i_ is expressed as

(3) $$C_{loss}=\sum_{i\in C}\mu_{i}q_{i}(1-\theta_{p}(t_{i}))$$

where µ_i_ is the unit value of the agricultural product.

Regarding the selection of the freshness decay parameter λp = 0.002 h^−1^: this value corresponds to the typical decay coefficient of Class II perishable agricultural products (fresh fruits/vegetables and dairy products) at the reference temperature (standard refrigeration temperature of 0–4 °C), and is referenced from experimentally measured decay rates of fresh products reported in the food cold chain logistics literature. The functional form of the temperature sensitivity factor η(T) is derived from the standard exponential form of the Arrhenius equation, with activation energy parameters referenced from published experimental data in the fresh agricultural product cold chain domain. The simulated values of the temperature-control failure factor β are set in the range of 1.5–3.0, referencing industry statistics on refrigeration equipment failure rates, to simulate varying degrees of temperature-control fault scenarios. It should be noted that the specific value of λp affects the weight allocation of the freshness objective in the optimization results, and different product categories (e.g., fresh-cut flowers, frozen meat) may have substantially different *λp* values. In practical applications, calibration should be performed by consulting literature or conducting experimental measurements for the specific product category.

### 2.3. Multi-Objective Optimization Formulation

To jointly optimize economic performance, service quality, and environmental sustainability, we define a four-objective optimization model. The first objective minimizes total logistics cost, which includes vehicle activation costs, fuel and refrigeration operating costs, soft time-window penalties, and cargo loss costs:

(4) $$\min f_{1}=\sum_{k\in K}F_{k}+\sum_{k\in K}\sum_{(i,j)\in E}(C_{fuel}FC_{ij}^{k}+C_{elec}EC_{ij}^{k})+\sum_{i\in C}P(t_{i})+C_{loss}$$

where F_k_ denotes the activation cost of vehicle k, and $EC_{ij}^{k}$ denotes fuel consumption and refrigeration electricity consumption on edge (i,j). p(t_i_) is the soft time-window penalty associated with deviations from the customer time window [ETi, LTi]. The second objective minimizes total delivery time to promote operational efficiency, defined as the total duration for all vehicles to complete their routes and return to the depot:

(5) $$\min f_{2}=\sum_{k\in K}\left(t_{end}^{k}-t_{start}^{k}\right)$$

The third objective minimizes total carbon emissions from both fuel combustion and refrigeration electricity use:

(6) $$\min f_{3}=w_{fuel}\sum_{k\in K}\sum_{(i,j)\in E}FC_{ij}^{k}+w_{elec}\sum_{k\in K}\sum_{(i,j)\in E}EC_{ij}^{k}$$

where w_fuel_ and w_elec_ are carbon emission factors for diesel and electricity in kg/L and kg/kWh, respectively. The fourth objective maximizes average terminal freshness as a direct proxy for customer satisfaction:

(7) $$\max f_{4}=\frac{1}{\left|C\right|}\sum_{i\in C}\theta_{p}(t_{i})$$

The model is subject to standard feasibility constraints in vehicle routes. Flow conservation ensures that each customer is served exactly once by a single vehicle. Capacity constraints enforce $\sum_{i\in R{\mathrm{oute}}_{k}}q_{i}\leq Q_{k}$ for all k∈K, where Q_k_ is the vehicle capacity. Time-window constraints impose a hard upper bound t_i_ ≤ LT_i_ + ∆_max_ allowing a limited maximum delay ∆_max_ while enforcing large penalties outside the soft time window through P(t_i_).

This formulation is a canonical NP-hard multi-objective combinatorial optimization problem [42]. Due to its inherent complexity, exact solvers are generally impractical at realistic scales, as they require immense computational resources that are not feasible for real-world applications. Moreover, a single deep reinforcement learning agent often struggles to manage the Pareto trade-off [43] across multiple conflicting objectives; such an agent typically relies on scalarized reward signals, which collapse multi-dimensional objectives into a single metric, thereby losing the ability to capture the true Pareto front. To address these challenges comprehensively, we therefore introduce the H-MODRL framework to approximate the optimal frontier for the ACCL-RP problem under dynamic disruptions. By leveraging a hierarchical structure and multi-objective optimization, this framework enables the agent to explore diverse trade-off strategies and generate a well-distributed set of near-Pareto-optimal solutions, providing a scalable and adaptive solution capable of responding to real-time environmental changes. This capability is particularly critical for ACCL-RP, where real-time responsiveness and balanced optimization across conflicting goals are essential for ensuring system reliability and efficiency in practice. Furthermore, by generating a diverse set of Pareto-optimal solutions, the framework equips practitioners with the flexibility to adapt their decisions based on varying real-time priorities and constraints.

## 3. Methodology: The H-MODRL Framework

### 3.1. Overview of H-MODRL

The H-MODRL framework is designed to systematically address multi-objective conflicts, real-time responsiveness, and cold-start limitations in dynamic agricultural cold chain logistics route planning. By coupling complementary optimization paradigms in a three-stage workflow, H-MODRL enables a coherent transition from rapid feasible-solution construction to high-quality multi-objective decision-making.

**Figure 1:**
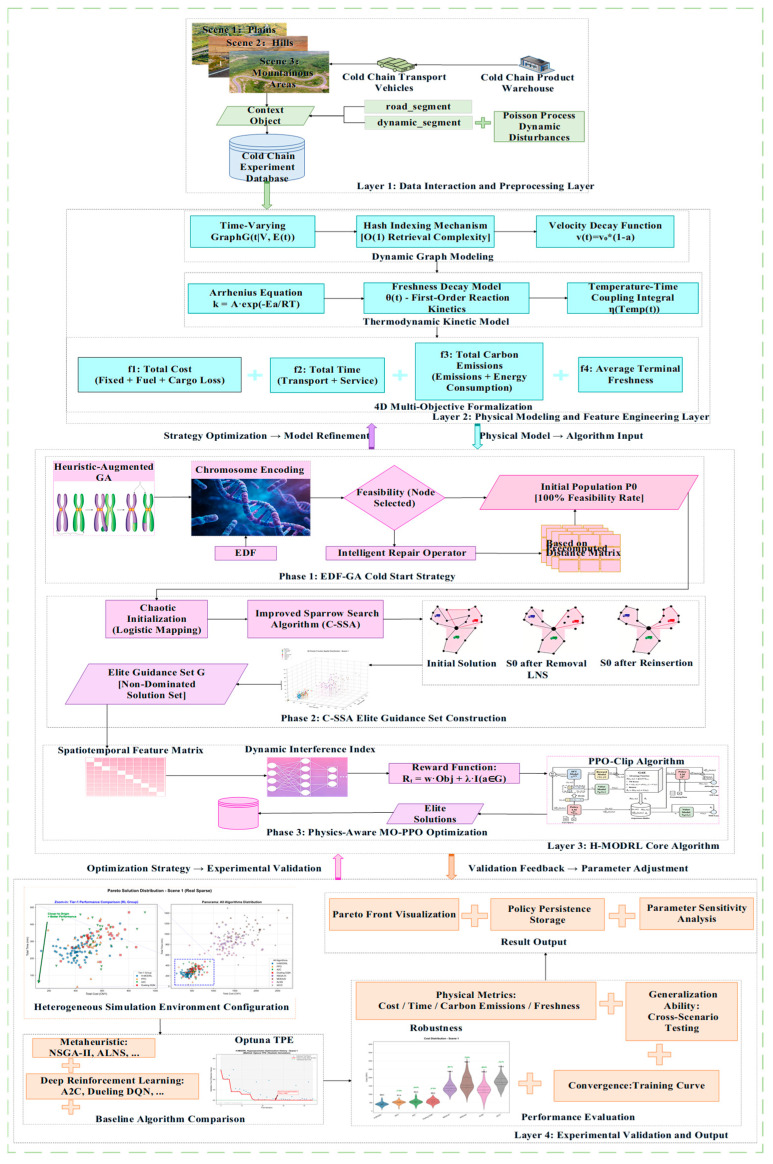
The framework of H-MODRL.

An overview of the framework is shown in Figure 1. The central idea is to combine the global exploration strength of metaheuristics with the fast inference and generalization capacity of deep reinforcement learning. The pipeline starts with an initialization stage, where an improved Genetic Algorithm (GA) augmented by the Earliest Deadline First (EDF) rule rapidly generates a population of feasible solutions that satisfy hard constraints such as vehicle capacity and time windows, thereby alleviating the cold-start difficulty commonly observed in DRL. The guidance stage then applies a Chaotic Sparrow Search Algorithm (C-SSA) that integrates Chaotic Mapping with a Large Neighborhood Search (LNS) operator to intensively refine the initial population and construct an Elite Guiding Set that covers the critical regions of the Pareto front. In the decision stage, the Elite Guiding Set is injected as prior knowledge into a multi-objective deep reinforcement learning model built on Proximal Policy Optimization (PPO). This model incorporates a physics-based reward function and an action-mask mechanism, enabling online, real-time responses to dynamic disruptions while outputting routing decisions that respect multi-objective trade-offs.

### 3.2. Genetic Algorithm Initialization Based on EDF

In Stage 1, we adopt binary encoding for the genetic algorithm (GA) to facilitate crossover and mutation. For a parameter in (L,U) encoded by k bits, let b = b_k_b_k−1_…b_1_ with b_i_∈{0,1}. The decoding directly maps b to x∈[L,U] as

(8) $$x=L+(\sum_{i=1}^{k}b_{i}2^{i-1})\frac{U-L}{2^{k}-1}$$

Selection: Individuals are scored by a positive fitness f_i_; fitness-proportionate selection uses

(9) $$P_{i}=\frac{f_{i}}{\sum_{k=1}^{N}f_{k}}$$

Heuristic-Augmented GA Initialization. This stage rapidly constructs a fully feasible initial population P_0_ under hard time-window constraints. Chromosome construction is biased by Earliest Deadline First (EDF): for unserved customers C, node i is sampled with $P\left(i\right)\propto {\left(LT_{i}\right)}^{-1}$. Infeasible offspring are corrected by a Smart Repair Operator that, using a precomputed distance matrix, greedily reinserts violating nodes into the feasible position with minimal added cost, ensuring 100% feasibility of P_0_ for warm-starting subsequent optimization.

This stage further targets the low exploration efficiency of DRL agents under stringent time-window constraints by rapidly constructing an initial population $P_{0}$ that satisfies all hard constraints. We develop a Heuristic-Augmented GA that injects an Earliest Deadline First (EDF) bias during chromosome construction. For the unserved customer set C, customer i is selected with probability Pi∝LTi^−1^, where LTi denotes the latest allowable service time. To handle infeasible offspring generated during evolution, we introduce a Smart Repair Operator. Using a precomputed distance matrix, nodes that violate constraints are greedily reinserted into feasible positions that incur the minimal additional cost.

Smart Repair Operator. To address capacity violations and time-window violations that may arise after crossover and mutation, a two-layer Smart Repair Operator is designed that restores infeasible solutions to feasibility while minimally altering the original chromosome structure. The repair operator consists of two layers:

1. **Capacity Violation Repair:** When the cumulative demand on a vehicle route in an offspring chromosome exceeds the vehicle capacity Qk, a subset of nodes is removed from that route. Nodes with tighter time windows (i.e., smaller LTi) are preferentially retained, while removed nodes are added to an unassigned set U.
2. **Time-Window Violation Repair:** When the arrival time at a node j exceeds its hard time-window upper bound LTj + Δmax, the algorithm first attempts to adjust the service order of that node within the current vehicle route. If no feasible position can be found in the current route, node j is moved to the unassigned set U and reassigned to another vehicle route with the largest time-window slack.
3. **Final Reassignment:** For all nodes in the unassigned set U, the precomputed APSP distance matrix is used to greedily insert each node into the feasible position that incurs the minimal additional cost (incremental distance plus time-window penalty increment) across all routes.

This repair mechanism guarantees a 100% feasibility rate for all individuals in P_0_, providing a robust warm-start foundation for subsequent optimization.

Component Contribution: The core contribution of GA-EDF initialization is to provide a 100% feasible warm-start population, sparing the subsequent DRL training from having to start from a random policy. Without this stage, the DRL agent would need to explore the enormous combinatorial path space from scratch under strict hard time-window constraints, making early training extremely inefficient; in the worst case, it might never discover any feasible solution. The EDF bias and the Smart Repair Operator jointly ensure the quality and feasibility of the initial population.

### 3.3. Chaotic Sparrow Search Guidance

Stage 2 uses the Chaotic Sparrow Search Algorithm (C-SSA) to construct an Elite Guiding Set that provides diverse, high-quality priors for the downstream policy network. The Sparrow Search Algorithm (SSA) is a swarm-intelligence optimizer that partitions the population into producers and scroungers. Producers possess stronger “resources” and primarily determine the global search direction, whereas scroungers follow producers and exploit nearby regions. A small proportion of individuals act as sentinels (warners): when predation risk is detected, the population switches from exploration to escape behavior, where boundary individuals move rapidly toward safer regions and central individuals perform stochastic movements to maintain cohesion. Roles are updated across iterations, allowing individuals to switch between producer and scrounger while keeping their overall proportions approximately constant. Resource-poor scroungers tend to explore more aggressively, and scroungers typically forage around producers, potentially competing for resources. Let the swarm consist of n sparrows in a d-dimensional search space. The population is represented as a position matrix:

(10) $$X=\left(\begin{array}{ccc} x_{11} & \ldots & x_{1d}\\ \vdots & \ddots & \vdots \\ x_{n1} & \ldots & x_{nd} \end{array}\right)$$

where x_ij_ denotes the j-th coordinate of sparrow i. Fitness values are computed by applying the objective function f to each individual:

(11) $$F_{X}=\left(\begin{array}{c} f\left(x_{11},x_{12},\ldots ,x_{1d}\right)\\ f\left(x_{21},x_{22},\ldots ,x_{2d}\right)\\ \vdots \\ f\left(x_{n1},x_{n2},\ldots ,x_{nd}\right) \end{array}\right)$$

During each iteration, producers with higher fitness obtain priority and explore a larger region, updating their positions as

(12) $$X_{i,j}^{t+1}={\{}_{X_{i,j}^{t}+Q\cdot L,if\;R_{2}\geq ST}^{X_{i,j}^{t}\cdot \exp(-\frac{i}{\alpha \cdot T_{\max}}),if\;R_{2}<ST}$$

where t is the iteration index, $\left.\alpha \in \right.$ (0,1] is a uniform random variable, $T_{max}$ is the maximum number of iterations, Q is a Gaussian random variable, L is a 1×d all-ones vector, $R_{2}\in$ [0, 1] is the warning value, and ST∈ [0.5, 1] is the safety threshold. When $R_{2}<ST$, the local environment is considered safe and the swarm performs broader exploration; when $R_{2}\geq ST$, predation risk is detected, and the swarm rapidly shifts toward safer regions. Scroungers update their positions by following producers, with the update rule:

(13) $$X_{i,j}^{t+1}={\{}_{X_{p}^{t+1}+\left|X_{i,j}^{t}-X_{p}^{t+1}\right|\cdot L\cdot A^{+},i\leq 0.5n}^{Q\cdot \exp(\frac{X_{worst}^{t}-X_{i,j}^{t}}{i^{2}}),i>0.5n}$$

where $X_{P}^{t+1}$ denotes the current best (producer) position at iteration t+1, $X_{\mathrm{worst}}^{t}$ denotes the current worst position at iteration t, L is a 1×d all-ones vector, and A is a 1×d vector whose elements are randomly set to 1 or −1 with $A^{+}=A^{T}(AA^{T}{)}^{-1}$. Individuals with i>n/2 are treated as poorer scroungers and are encouraged to move away to search for new regions, whereas those with i≤n/2 perform local foraging around $X_{P}^{t+1}$.

Sentinels comprise approximately 10–20% of the population and broadcast warning signals; their update rule is:

(14) $$X_{D,i,j}^{t+1}={\{}_{X_{D,i,j}^{t}+K\cdot \left|\frac{X_{D,i,j}^{t}-X_{L}}{(fi-fw)+\varepsilon^{\prime }}\right|,f_{i}\leq f_{g}}^{X_{B}^{t}+\beta \cdot \left|X_{D,i,j}^{t}-X_{B}^{t}\right|,f_{i}>f_{g}}$$

where X_B_ is the current global best position, f_i_ is the fitness of sentinel i, f_g,_ and f_w_ are the best and worst fitness values in the current population, β is a Gaussian random variable with mean 0 and variance 1, K∈[−1, 1] is a random scalar, and ε is a small constant preventing division by zero. This mechanism drives boundary individuals to move toward safety while steering central individuals to regroup, thereby improving robustness under risk.

To improve population diversity at initialization, we use the Logistic chaotic map, a widely used chaotic mapping with a simple form:

(15) $$Y_{n+1}=aY_{n}(1-Y_{n})$$

where Y_n_∈[0, 1] and a∈[0, 4] is the control parameter. We construct the Elite Guiding Set G by leveraging swarm-level global search to avoid local optima and to obtain a diverse set of high-quality non-dominated solutions. Specifically, C-SSA enhances SSA in two aspects. First, Chaotic Initialization uses the Logistic chaotic map to initialize sparrow positions, improving ergodicity and coverage of the search space. Second, LNS-based scouting embeds a Large Neighborhood Search (LNS) operator into the sentinel (scout) behavior. A Random Destroy operator removes k% of route segments, and a Regret Repair operator reconstructs the partial solution. This destroy-and-repair mechanism helps the search escape the strong attraction basins of local minima and yields a non-dominated solution set E* that better approximates the Pareto front.

This algorithm enhances the standard SSA in two key aspects. (i) Chaotic Initialization (Step 1): The Logistic chaotic map replaces uniform random initialization, improving the ergodicity and coverage of the search space to avoid premature convergence to local optima. (ii) LNS-Enhanced Sentinel Search (Step 7b): The Large Neighborhood Search operator from operations research is embedded into the sentinel position update mechanism, replacing the original random-walk strategy. Through the iterative “destroy-repair” process, each solution undergoes deep optimization, significantly enhancing both local exploitation capability and local-optimum escape ability. The Time-Hegemony-Guided Greedy Repair operator prioritizes routes with the largest time-window slack during node reinsertion, strengthening the algorithm’s capability to optimize delivery timeliness.

Component Contribution: The core contribution of the C-SSA-LNS guidance stage is to address the insufficient Pareto-front coverage problem in scalarized multi-objective RL. The Elite Guiding Set G is essentially a collection of finely optimized non-dominated solutions covering key regions of the Pareto front (e.g., cost-prioritized, time-prioritized, and balanced solutions). During PPO training, the guidance term I(at∈G) provides positive reinforcement when the agent’s actions match fragments of elite solutions. This imitation-style guidance mechanism significantly narrows the effective exploration space and accelerates convergence toward high-quality Pareto regions.

### 3.4. Physics-Based Multi-Objective Proximal Policy Optimization

This stage constitutes the core decision engine of H-MODRL. We adopt the Actor-Critic architecture and tightly couple policy learning with physics-based modeling, drawing inspiration from the wind-field correction and surrogate-divergence optimization strategy proposed by Gan et al. [44]. The design focuses on three aspects: a disruption-aware state representation with constant-time querying, a physics-based reward that provides multi-objective learning signals, and a stable PPO-Clip update rule with solution persistence.

State space and dynamic indexing. To handle dynamic disruptions, the state $S_{t}$ extends standard routing descriptors (e.g., vehicle location and remaining capacity) with precomputed spatio-temporal features derived from All-Pairs Shortest Paths (APSP). We use the Floyd–Warshall algorithm to compute shortest-path distances between all pairs of nodes. Given a directed graph G = (V,E) and an initial weight matrix w[i][j], the edge-weight definition is

(16) $$W_{ij}={\{}_{\infty ,(i,j)\notin E}^{weight(i,j),(i,j)\in E}$$

Let D_ijk_ denote the shortest path length from i to j using only nodes in {1, …, k} as intermediate nodes. The dynamic-programming recursion is

(17) $$D_{ijk}=\min(D_{ij(k-1)},D_{ik(k-1)}+D_{kj(K-1)})$$

Time Complexity of Online Decision-Making: The complete online re-planning workflow decomposes into three serial stages, each with distinct complexity characteristics:

1. Disruption Event Query—O(1): When a dynamic disruption δk occurs, the system identifies the affected road segment Iij and retrieves its parameters (α, β, τstart, τend) in constant time via the pre-built hash index Ωidx, which maps each road segment to its current disruption record. This index was constructed during environment initialization (Algorithm 1, Step 1), and lookup requires only a single hash-table access regardless of network scale.
2. Fast State Update—O(|V|^2^) matrix lookup: Based on the precomputed APSP matrix (initialized once via the Floyd–Warshall algorithm with O(|V|^3^) complexity), the online phase requires only matrix lookups and path concatenation. For moderate-scale networks (|V| = 50–100 as in our three scenarios), these operations complete at the millisecond level.
3. Policy Network Inference—network-size dependent: The forward propagation of the PPO policy network constitutes the dominant latency component. For the current architecture (3-layer MLP with 256 hidden units per layer), a single forward pass takes approximately 0.5–2 ms on the GPU hardware specified in Section 4.1.

**Algorithm 1: C-SSA Elite Guiding Set Construction Algorithm with LNS and Chaotic Mapping**
Input: Initial feasible population P from EDF-GA, multi-objective function F, population size n, maximum iterations Tmax, safety threshold ST, destruction ratio k Output: Pareto-optimal Elite Guiding Set E*.
Initialization: Set t = 0. Generate initial sparrow position matrix X using Logistic chaotic map Yn + 1 = aYn(1 − Yn). Apply the Smart Repair Operator to ensure 100% feasibility.Compute multi-objective function values F(X) and fitness for all individuals.while t < Tmax doClassify roles: Top 15% by fitness → Producers, remaining 85% → Scroungers, randomly select 10% → Sentinels.Update producer positions via Equation (12). Apply the Smart Repair Operator after the update.Update scrounger positions via Equation (13). Apply the Smart Repair Operator after the update.Update sentinel positions: Determine type (boundary/center). Center sentinels execute LNS “Destroy-Repair” operator:7a. Random Destroy operator: Randomly remove k% of route segment nodes.7b. Time-Hegemony-Guided Greedy Repair operator: Reinsert removed nodes into optimal positions, prioritizing routes with largest time-window slack.Constraint verification and repair: Apply the Smart Repair Operator to all individuals. Remove infeasible individuals; refill with chaotic-initialized feasible solutions.Compute multi-objective function values, Pareto ranks, and crowding distances.Elitism preservation: Retain all Rank = 1 non-dominated solutions for the next generation. Fill remaining slots by descending crowding distance to maintain population size n.Global escape check: If the global best is not updated for 5 consecutive generations, apply LNS operator to 50% of non-optimal individuals for global neighborhood search.t = t + 1end whileElite guiding set selection: Perform fast non-dominated sorting on the final population. Select Rank = 1 non-dominated solutions to construct Elite Guiding Set E* (size 50~100).Output Elite Guiding Set E* and the corresponding (st, at) expert trajectory pairs.

Thus, the total online decision latency is controlled within a few milliseconds, well within the real-time requirements of practical cold chain dispatching.

It is important to distinguish between optimization quality and online inference latency when interpreting the above online response performance. The All-Pairs Shortest Paths (APSP) precomputation and the O(1) hash-based disruption indexing mechanism (Omega_idx) are engineering accelerations implemented specifically for H-MODRL to enable rapid state updates during online inference. Among the seven baseline algorithms evaluated in this study, the three deep reinforcement learning baselines (A2C, Dueling DQN, and Vanilla PPO) also possess the capability of offline training followed by online millisecond-level inference through their respective policy/value networks. However, these RL baselines do not employ the APSP precomputation and hash-based disruption indexing accelerations; instead, they perform distance computation and state updates using their standard procedures (e.g., on-the-fly shortest-path recomputation upon each disruption event), which may incur higher per-event latency. The four heuristic baselines (NSGA-III, MOEA/D, ALNS, and IACO) operate on a fundamentally different paradigm: when a dynamic disruption occurs, they must re-execute the entire evolutionary or neighborhood search process—typically requiring hundreds to thousands of population iterations, with single optimization runs lasting from tens of seconds to several minutes—rendering them inherently unsuitable for real-time replanning scenarios. Therefore, the online response time advantage of H-MODRL should be attributed partly to (i) its dedicated APSP-and-hash-index acceleration infrastructure, and partly to (ii) the separation of expensive offline training (Stages 1–3) from lightweight online inference (policy network forward pass only). Meanwhile, the routing quality advantage—as measured by the four objective metrics (total cost, delivery time, carbon emissions, and terminal freshness)—stems from the hybrid algorithmic design (GA-EDF warm-start providing 100% feasible initial populations, C-SSA-LNS elite guidance covering key Pareto-front regions, and physics-based MO-PPO decision-making with Arrhenius-embedded reward shaping). All methods are evaluated on identical problem instances against the same four objective functions, with all baselines having undergone grid-search hyperparameter optimization for fair comparison; therefore, the per-event planning quality comparison remains valid and unbiased. However, a head-to-head comparison of online latency between H-MODRL and the baselines would require all methods to be instrumented with equivalent acceleration infrastructure (APSP precomputation and hash-based indexing), which falls outside the scope of the present study.

The resulting disruption-aware state is defined as:

(18) $$S_{t}=[Loc_{norm},Load_{pct},Time_{remain},\Omega_{idx}(t),Mask_{t}]$$

Physics-based reward shaping. The instantaneous reward R_t_ is derived directly from the physics-based models introduced in Chapter 2 to provide scalarized multi-objective feedback:

(19) $$R_{t}=-(w_{1}\Delta C_{trans}+w_{2}\Delta T_{total}+w_{3}\Delta E_{carbon}+w_{4}\Delta Q_{loss})+\lambda_{guide}\cdot (a_{t}\in G)$$

where ΔQloss denotes the real-time cargo loss computed from the Arrhenius-based freshness decay model. The four scalarization weights {w1, w2, w3, w4} control the relative emphasis placed on each objective in the compound reward signal. In all experiments reported in this paper, we adopt the default balanced setting w1 = 0.35 (cost), w2 = 0.15 (time), w3 = 0.25 (carbon emissions), and w4 = 0.25 (freshness). These values were determined after per-objective min-max normalization so that each term contributes on a comparable scale, and they follow common practice in the multi-objective cold chain logistics optimization literature. We note that weight selection critically shapes the Pareto trade-off behavior, and different application scenarios (e.g., cost-sensitive versus freshness-prioritized dispatching) may warrant different weight configurations. A systematic sensitivity analysis of these weights is identified as an important direction for future work.

The guidance term I(at∈G) provides positive reinforcement when the agent’s chosen action at time t matches a high-quality route fragment contained in the Elite Guiding Set G constructed during Stage 2, thereby coupling imitation-style guidance with standard reinforcement learning.

Policy update and persistence. We update the policy using PPO-Clip, which constrains policy changes to improve stability and prevent destructive updates. The clipped surrogate objective is:

(20) $$\;\underset{\theta }{max}E_{s∼\rho^{\pi_{k}},|a∼\pi_{k}\left(\cdot |s\right)}\left[min\left(r_{\theta }\left(s,a\right)\right.A^{\pi_{\theta_{k}}}\left(s,a\right),|clip\left(r_{\theta }\left(s,a\right),|1-\varepsilon ,|1+\varepsilon \right)A^{\pi_{\theta_{k}}}\left(s,a\right))\right]$$

where $r_{\theta }\left(s,a\right)=\pi_{\theta }\left(a|s\right)/\pi_{\theta_{k}}\left(a|s\right)$ denotes the probability ratio between new and old policies, $A^{\pi_{\theta_{k}}}\left(s,a\right)$ is the advantage under the behavior policy π_θk_, and ε defines the clipping range [1 − ε, 1 + ε]. After training, the obtained Pareto solution set is subjected to a second-round physics-based validation, and the final Elite Solutions are persisted to a database for downstream visual analytics.

Component Contribution: The core role of MO-PPO is not to replace heuristic search but to endow the model with online adaptive capability in response to dynamic disruptions. GA-EDF and C-SSA-LNS produce high-quality static solution sets during the offline phase, but they inherently lack real-time responsiveness—when a dynamic disruption occurs (e.g., road closure, congestion, temperature-control fault), the entire evolutionary search process must be re-executed, incurring a prohibitively high computational cost. Once MO-PPO training is complete, the policy network can instantly respond to newly emerging dynamic states at a fixed inference cost, which is a core capability that heuristic methods cannot achieve. The role of the Elite Guiding Set is to accelerate and stabilize training convergence, not to replace the online inference function. The training-inference separation design cleanly decouples expensive offline training (including the three-stage pipeline) from lightweight online deployment (requiring only policy inference). Furthermore, the physics-based reward function ensures that the optimization direction is consistent with Arrhenius freshness-decay kinetics, preventing purely data-driven optimization from producing routing plans with physical meaning mismatch.

## 4. Results

### 4.1. Experimental Setup

Dataset Construction. We construct a heterogeneous terrain simulation dataset that better reflects Agricultural Cold Chain Logistics (ACCL) using real geographic information from China. The high-fidelity environment follows the physics-aware modelling paradigm proposed by Wu et al. [45]. The dataset construction strictly adheres to three principles (see Table 1):

1. Physical Fidelity: The state space, reward function, and optimization logic of the algorithm are tightly coupled to the agricultural cold chain physical model, with freshness-decay kinetics, fuel/refrigeration carbon emission mechanisms, and rural road network time-varying characteristics directly embedded in the algorithm core.
2. Scenario Gradient: Based on the general classification standard of rural geographic environments in China, the three scenarios form a clear gradient progression in road network density (2.1 → 0.95 → 0.45 km/km^2^), terrain complexity, and dynamic disturbance intensity, achieving full-spectrum coverage from basic operating conditions to extreme conditions.
3. Engineering Compatibility: The customer node scale, refrigerated vehicle performance parameters, agricultural product category structure, and time-window constraint rules are all strictly matched to the actual operating specifications of county-level agricultural cold chain enterprises in China, ensuring that experimental results have direct engineering deployment reference value.

Static road network topology data were sourced from multiple authoritative Chinese geographic information platforms: the National Platform for Common Geospatial Information Services (Tianditu) and the National Geographic Information Resources Directory Service System provided rural vector road network and geographic feature data; the Resource and Environment Science and Data Center of the Chinese Academy of Sciences provided 30 m-resolution Digital Elevation Model (DEM) terrain data for accurate slope and elevation calibration. Road network structures were extracted for three representative Chinese counties spanning distinct geographic regimes, with the following detailed configurations:

- Scene 1 (Plain): A plain agricultural county in a North China Plain peri-urban area, geographic coverage ~50 km^2^, 84 customer nodes, single distribution center, road network density ~2.1 km/km^2^ (regular grid topology), paved cement road ratio ~90%, unpaved dirt road ratio ~10%, mountain/hill climbing segments ~0%, average segment distance ~1.2 km, baseline speed limit 60 km/h.
- Scene 2 (Hilly): A hilly county in a shallow-hill agricultural area (e.g., Sichuan Basin periphery), geographic coverage ~120 km^2^, 68 customer nodes, single distribution center, road network density ~0.95 km/km^2^ (random layout topology), paved cement road ratio ~60%, unpaved dirt road ratio ~30%, mountain/hill climbing segments ~10%, average segment distance ~3.5 km, baseline speed limit 40 km/h.
- Scene 3 (Mountainous): A mountainous county in a remote high-altitude area (e.g., Yunnan-Guizhou Plateau region), geographic coverage ~280 km^2^, 50 customer nodes, single distribution center, road network density ~0.45 km/km^2^ (sparse tree-like topology), paved cement road ratio ~30%, unpaved dirt road ratio ~50%, mountain/hill climbing segments ~20%, average segment distance ~7.8 km, baseline speed limit 30 km/h.

The road network density ranges (2.0–2.5, 0.8–1.2, and 0.3–0.6 km/km^2^ for plain, hilly, and mountainous areas respectively), rural road pavement rates, and speed limits are consistent with official data published by the Ministry of Natural Resources and the Ministry of Transport of China. Customer node scale and spatial distribution are referenced from the Alibaba Tianchi Cold Chain Logistics open dataset to match the population distribution characteristics of Chinese rural areas.

Dynamic disturbance feature parameters were calibrated as follows. Disruption event arrival follows a homogeneous Poisson process. The congestion frequency lambda_dist is set to 0.2 events/h (Scene 1), 0.5 events/h (Scene 2), and 1.0 events/h (Scene 3). The speed attenuation factor ranges from 20–30% (Scene 1), 30–50% (Scene 2), to 50–80% (Scene 3), reflecting progressively severe capacity reduction on narrower mountain roads. The temperature-control failure factor beta ranges from 1.5 to 3.0 across all scenarios. Four categories of dynamic disruptions are modeled, with detailed occurrence probabilities and duration distributions calibrated from historical data:

(i) Traffic congestion: occurrence governed by Poisson process with lambda_dist, duration follows Lognormal distribution (hours), congestion intensity proportional to speed attenuation factor alpha. (ii) Road temporary closure: occurrence probabilities 2% (Scene 1), 8% (Scene 2), 15% (Scene 3); duration follows Uniform(2, 6) hours; affected road segment set to impassable. (iii) Severe weather: occurrence probabilities 5% (Scene 1), 15% (Scene 2), 30% (Scene 3); duration follows Uniform(4, 12) hours; weather impact coefficient 1.2 (rain) to 1.5 (snow); refrigeration energy consumption proportional to squared temperature difference. (iv) Temperature-control equipment failure: occurrence probabilities 1% (Scene 1), 3% (Scene 2), 5% (Scene 3); duration follows Uniform(0.5, 2) hours; temperature drift coefficient beta = 1.5–3.0; freshness decay rate positively correlated with beta.

All dynamic parameters were calibrated using long-series meteorological measurement data from the National Meteorological Science Data Center of the China Meteorological Administration and historical rural road traffic condition data from Gaode Map (Amap) and Baidu Map open platforms. Traffic congestion parameters follow the standard traffic engineering model combining Poisson process with Lognormal duration distribution, referenced from domestic rural road empirical statistics. Road closure parameters are referenced from Ministry of Transport rural road maintenance data. Severe weather parameters are referenced from CMA rural meteorological statistics. Temperature-control equipment failure parameters are referenced from cold chain logistics vehicle maintenance data.

Customer demand parameters were derived from publicly available agricultural product circulation data sources, including the Alibaba Tianchi Cold Chain Logistics open dataset and the HeyWhale (Kesci) community’s domestic cold chain enterprise de-identified operational datasets, as well as the China Cold Chain Logistics Development Report (CFLP, 2024). Key business parameters are configured as follows: vehicle rated payload Q_k = 5000 kg (corresponding to the Chinese standard 4.2 m refrigerated truck); vehicle rated volume = 20 m^3^.

Diesel carbon emission factor w_fuel = 2.63 kg/L; electricity carbon emission factor w_elec = 0.581 kg/kWh; freshness decay coefficient lambda_p = 0.002 h^−1^ (corresponding to Class II perishable agricultural products—fresh fruits/vegetables and dairy products—at standard refrigeration temperature 0–4 degrees C, referenced from experimentally measured decay rates in food cold chain logistics literature); initial freshness theta_0 = 1.0.

Agricultural product category composition varies by scenario: Scene 1 (leafy vegetables 60%, meat 30%, aquatic products 10%), Scene 2 (leafy vegetables 50%, meat 35%, aquatic products 15%), Scene 3 (leafy vegetables 40%, meat 40%, aquatic products 20%). Time-window constraints are Scene 1: soft time window, tolerance +/− 30 min; Scene 2: soft time window, tolerance +/− 20 min; Scene 3: hard time window, tolerance +/− 10 min. Average demand per node: 300 kg (Scene 1), 500 kg (Scene 2), 800 kg (Scene 3). All business parameters are configured to match the actual operational specifications of county-level agricultural cold chain enterprises in China. The complete parameter configuration for all three scenarios is provided in Table 1 and Table 2.

Regarding data availability: the complete simulation environment uses multiple third-party data sources (Tianditu road networks, CMA meteorological measurements, Amap/Baidu traffic conditions, and Alibaba Tianchi/HeyWhale logistics datasets). These raw third-party data are subject to their respective usage terms and licensing agreements and cannot be redistributed as part of this publication. The specific parameter configurations for reproducing the three simulation scenarios are fully documented in Table 1 and Table 2, and the disruption-generation rules are mathematically specified in Section 2.1 and Section 4.1. Additional implementation details may be obtained from the corresponding author upon reasonable request, subject to third-party data usage restrictions. The development of a publicly available, standardized multi-instance benchmark dataset for agricultural cold chain logistics optimization is identified as an important direction for community-wide collaborative effort.

Experimental Settings. The physical environment parameters are configured as follows. Each vehicle is constrained by a payload capacity of Q=5000 kg. The carbon emission factors are specified as $\xi_{\mathrm{fuel}}=2.63\;\mathrm{kg}/L\;$for diesel combustion and $\xi_{\mathrm{elec}}=0.58\;\mathrm{kg}/\mathrm{kWh}$ for electricity consumption. Regarding perishable goods, two high-value, temperature-sensitive commodities are selected as representative cargo. The freshness decay coefficient is defined as $\lambda_{p}=0.002\;h^{-1}$, with an initial freshness level of $\theta_{0}=1.0$. The hyperparameters for the proposed H-MODRL algorithm are detailed in Table 2. To ensure a fair comparison, all baseline methods were optimized using a grid search approach.

Baseline Algorithms. To evaluate H-MODRL in terms of multi-objective trade-off quality, dynamic responsiveness, and the structural properties of the solution set, we benchmark our approach against seven representative baselines spanning classical metaheuristics and deep reinforcement learning (DRL).

The heuristic group includes the Adaptive Large Neighborhood Search (ALNS), a widely adopted industrial-grade solver for the Vehicle Routing Problem (VRP) that explores the solution space by adaptively reweighting multiple destroy and repair operators. We follow the standard framework established by Ropke and Pisinger, incorporating a time-window-aware Shaw removal strategy to serve as a robust reference. IACO is an Ant Colony Optimization (ACO) variant for the VRP that models pheromone-based search and adopts a max-min ant system strategy to mitigate premature convergence [46]. NSGA-III is a canonical evolutionary algorithm for many-objective optimization that maintains population diversity via reference-point-based selection rather than crowding distance. Additionally, MOEA/D decomposes a multi-objective problem into scalar subproblems and optimizes them jointly [47].

The DRL group includes A2C, a classic synchronous on-policy policy-gradient method [48]. We adopt the A2C and PPO algorithms for coverage path planning with grid-based state encoding as reported by Garrido-Castañeda et al. [49], representing a strong on-policy baseline in this domain. Dueling DQN is a value-based off-policy method that separates state value and advantage estimation [50]. For this, we follow the improved Dueling Double Deep Q-Network (D3QN) with Prioritized Experience Replay (PER) proposed by Gök [51], which optimizes path planning in environments with static and dynamic obstacles to rigorously test adaptability. Finally, Vanilla PPO refers to a standard PPO model lacking the Elite Guiding Set and physics-based reward shaping; we utilize it as a direct baseline to quantify the marginal contributions of the proposed hybrid-evolution mechanism and physics-based modules.

Fairness of Baseline Comparison. To ensure a rigorous and fair comparison, the experimental setup adheres to the following principles: (1) All methods share an identical precomputed All-Pairs Shortest Path (APSP) distance matrix, which serves as the environmental infrastructure layer. They also operate within the same physics-based simulation environment, incorporating standardized freshness decay models, carbon emission calculators, and disruption event generators. (2) Engineering acceleration scope: The All-Pairs Shortest Paths (APSP) precomputation and the O(1) hash-based disruption index Omega_idx are engineering accelerations implemented specifically for H-MODRL to enable rapid state updates during online inference. Among the baseline methods, the three deep reinforcement learning algorithms (A2C, Dueling DQN, and Vanilla PPO) also possess offline-training-online-inference capability through their respective policy/value networks; however, they do not employ these precomputed acceleration structures, instead using their standard distance computation and state update procedures. The four heuristic baselines (NSGA-III, MOEA/D, ALNS, and IACO) operate on an iterative re-optimization paradigm that fundamentally cannot support real-time replanning. Consequently, while all methods are evaluated on identical problem instances against the same four objective metrics—ensuring fairness of optimization quality comparison—their underlying online deployment infrastructures differ. Readers should interpret the online response performance of H-MODRL as reflecting the combined effect of its hybrid algorithmic design (Stages 1–3) and its dedicated engineering accelerations (APSP and hash index), as detailed in Section 3.4. (3) All baseline methods were optimized via a grid search approach, ensuring comparable tuning efforts across their respective hyperparameter spaces.

Evaluation Metrics and Implementation Details. We assess algorithmic performance from two complementary perspectives. First, convergence behavior is evaluated via 2D/3D Pareto-front visualization and dominance analysis, focusing on both the spread of the non-dominated set and its proximity to the ideal frontier. Second, operational utility is quantified using four objective functions: $f_{1}(totalcost)$, $f_{2}(totaldeliverytime)$, $f_{3}(totalcarbonemissions)$, and $f_{4}(averageterminalfreshness)$. All experiments were conducted on a workstation equipped with an NVIDIA GeForce RTX 4090 GPU (24 GB) and an Intel Core i9-13900K CPU. To account for the greater number of stochastic sources (i.e., a three-stage stochastic process) inherent to the proposed method, H-MODRL was executed for 70 independent runs per experimental setting to ensure stable statistical estimates. In contrast, each baseline was executed for 30 runs. All reported results are based on the averaged values across these independent runs.

Remarks on Statistical Hypothesis Testing. We clarify that formal statistical hypothesis testing (e.g., the Wilcoxon rank-sum test or paired t-test with multiple comparison correction) is not performed in this study. The primary reason concerns the structure of the evaluation dataset. Unlike standard optimization benchmark suites—such as the Solomon or Gehring–Homberger instances for vehicle routing, or the CEC/ZDT/DTLZ suites for multi-objective optimization—which provide tens to hundreds of problem instances per difficulty level, this study employs a single custom-constructed heterogeneous terrain dataset. The three scenarios (plain, hilly, mountainous) are intentionally designed as a difficulty gradient with systematically varying road-network density (2.1 → 0.95 → 0.45 km/km^2^), geographic scale (50 → 120 → 280 km^2^), and disturbance frequency (low → medium → high). They do not constitute independent, identically distributed (i.i.d.) samples drawn from a common population, which is a prerequisite for the valid application of rank-based or parametric statistical tests across scenarios. Pooling per-run metric values from qualitatively distinct geographic regimes would violate the exchangeability assumption and produce test statistics of questionable interpretability.

Furthermore, to the best of our knowledge, no publicly available benchmark dataset currently exists for agricultural cold chain logistics that simultaneously integrates Arrhenius-based freshness-decay kinetics, full life-cycle carbon emission modeling, hard time-window constraints, and stochastic dynamic disruption modeling at the county operational scale. This absence of a standardized multi-instance benchmark makes cross-dataset statistical validation infeasible at the present stage.

As an alternative, this study provides the following multi-faceted evidence to assess performance reliability:

(1) Adequate independent runs: H-MODRL was executed for 70 independent runs per scenario, and each baseline for 30 runs, ensuring stable empirical estimates of the mean and variance. The larger run count for H-MODRL reflects the additional stochasticity introduced by its three-stage pipeline.

(2) Full distributional disclosure: The violin plots presented in Section 4.2, Section 4.3 and Section 4.4 present the complete empirical distribution (median, interquartile range, density shape, and outlier points) of each method on each metric, rather than reducing performance to a single scalar. Across all three scenarios and four objectives, H-MODRL yields the most compact distributions with the narrowest interquartile ranges.

(3) Pareto-front dominance: In multi-objective optimization, the Pareto dominance relation provides a rigorous, assumption-free comparison criterion. The 2D/3D Pareto-front visualizations presented in the subsequent sections demonstrate that H-MODRL’s non-dominated set consistently anchors near the ideal point, and no competing method produces any solution that Pareto-dominates an H-MODRL solution in the four-objective space.

(4) Cross-scenario consistency: H-MODRL achieves the best mean value on all four objectives in all three scenarios, with improvements over the strongest DRL baseline (Vanilla PPO) ranging from 13.4% to 33.6% across metrics and scenarios. This consistent ranking across qualitatively different terrain conditions provides evidence that the performance advantage is not an artifact of a particular scenario configuration.

We acknowledge that formal hypothesis testing would further strengthen the statistical rigor and plan to incorporate it in future work, contingent on the availability of suitable multi-instance benchmark datasets for agricultural cold chain logistics.

### 4.2. Full-Dimensional Performance Analysis in the Plain Scenario

**Figure 2:**
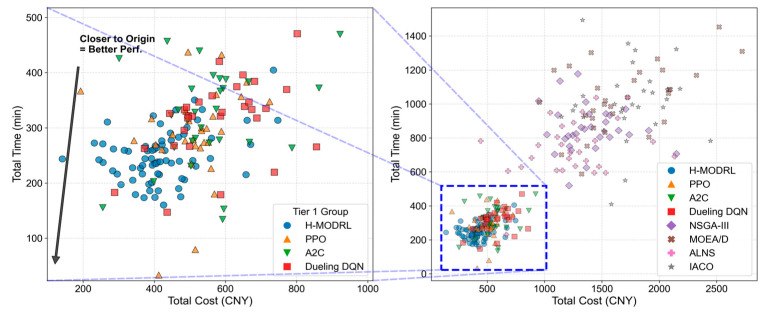
Pareto solution distribution—Scenario 1.

**Figure 3:**
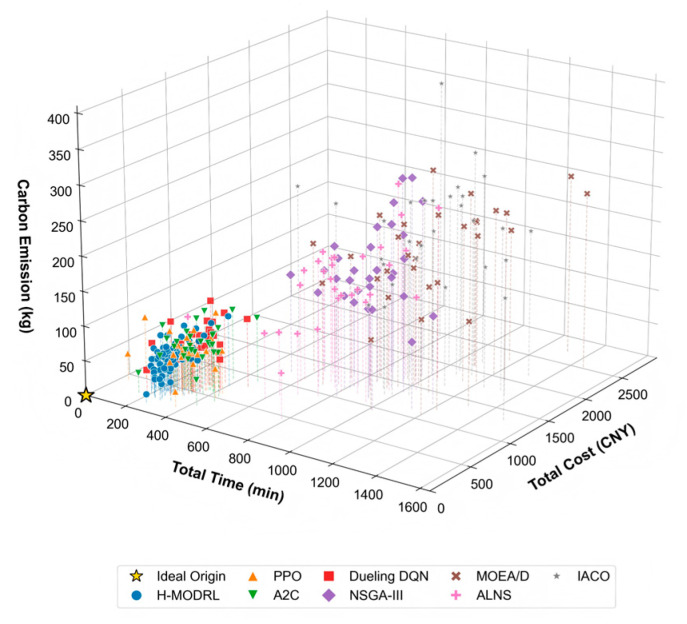
3D Pareto front spatial distribution—Scenario 1.

The Pareto front visualizations (Figure 2 and Figure 3) demonstrate that H-MODRL consistently dominates the objective space. Its non-dominated set converges closely to the ideal point and forms a compact cluster, indicating a superior trade-off among total cost, total delivery time, and carbon emissions while successfully preserving commodity freshness. In contrast, although the DRL baselines exhibit competitive convergence, their resulting fronts remain ultimately suboptimal. Their solutions predominantly lie on the outer envelope of the H-MODRL set and are strictly dominated within the cost–time projection, reflecting a limited capacity to navigate the optimal boundary under four-dimensional objective conflicts. Furthermore, the heuristic baselines perform substantially worse, yielding sparse solutions distributed across a distinctly inferior region characterized by high costs and extended delivery times. This underperformance highlights their susceptibility to local optima when operating under large-scale, discrete constraints and highly dynamic conditions.

**Figure 4:**
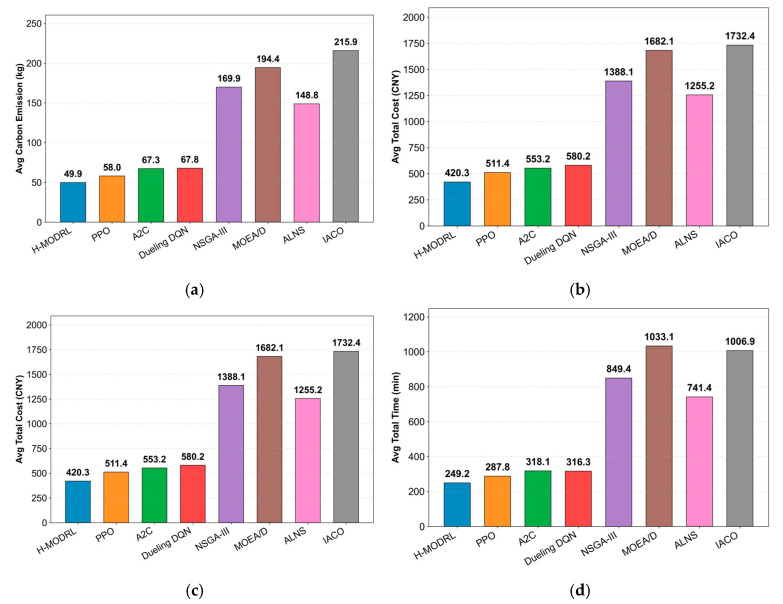
Comparison of multi-objective performance in Scenario 1. (**a**) Average total cost; (**b**) Average carbon emissions; (**c**) Average freshness; (**d**) Average total time.

This superior performance directly translates into consistent improvements across all four operational objectives (Figure 4a–d). Specifically, H-MODRL achieves the optimal mean performance, yielding an average total cost of 420.3 CNY, total carbon emissions of 49.9 kg, an average terminal freshness level of 0.958, and a total delivery time of 249.2 min. Compared to the strongest RL baseline (Vanilla PPO), H-MODRL reduces total cost and emissions by 17.8% and 13.9%, respectively, enhances time efficiency by 13.4%, and maintains a marginal advantage of approximately 1% in freshness. Furthermore, when contrasted with the weakest heuristic baseline (IACO), H-MODRL delivers drastic reductions of 75.7% in cost, 76.9% in emissions, and 75.9% in delivery time, alongside a substantial 17.0–19.5% improvement in commodity freshness.

**Figure 5:**
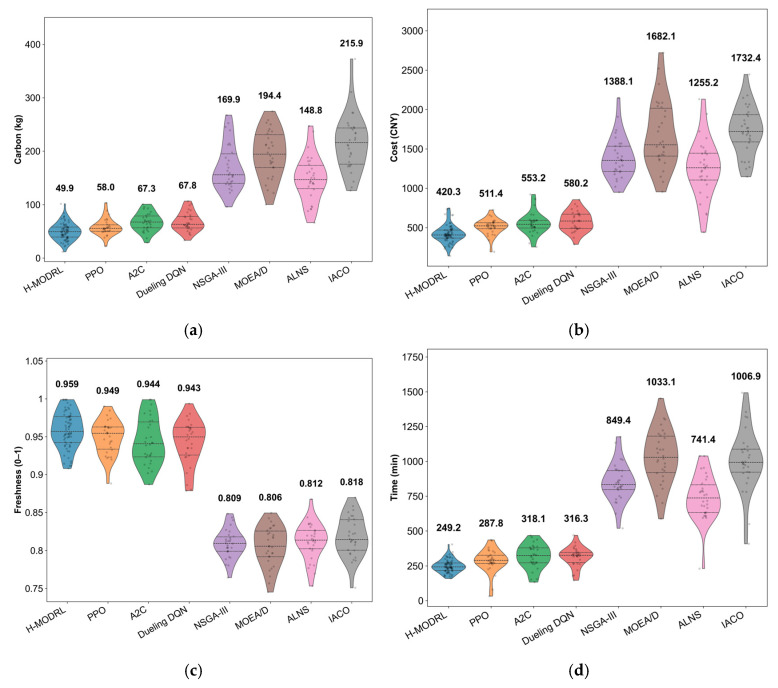
Distribution of performance indicators in Scenario 1. (**a**) Cost; (**b**) Carbon emissions; (**c**) Freshness; (**d**) Time.

The violin plots (Figure 5a–d) further substantiate the distributional robustness of the proposed method. Across all evaluation metrics, H-MODRL exhibits highly compact distributions characterized by minimal variance and an absence of pronounced heavy tails. In contrast, the RL baselines display wider spreads with occasional outliers representing low freshness or excessive delivery times, whereas the heuristic methods exhibit highly dispersed, heavy-tailed distributions. Collectively, these empirical findings demonstrate that H-MODRL not only advances the Pareto frontier within dense plain networks but also guarantees stable, low-variance performance under stochasticity, thereby validating its engineering reliability for real-world ACCL-RP.

### 4.3. Robustness Analysis in Hilly Terrain

**Figure 6:**
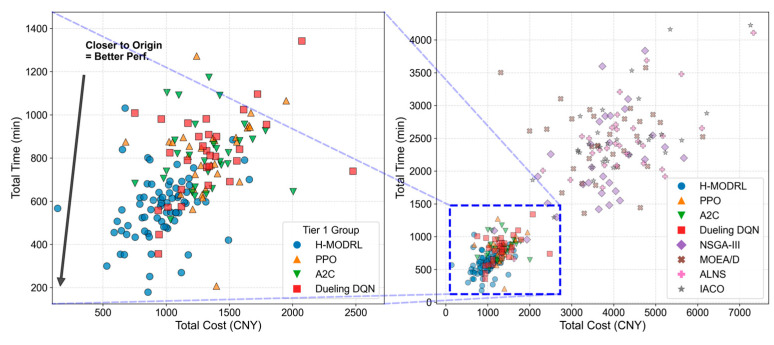
Pareto solution distribution—Scenario 2.

**Figure 7:**
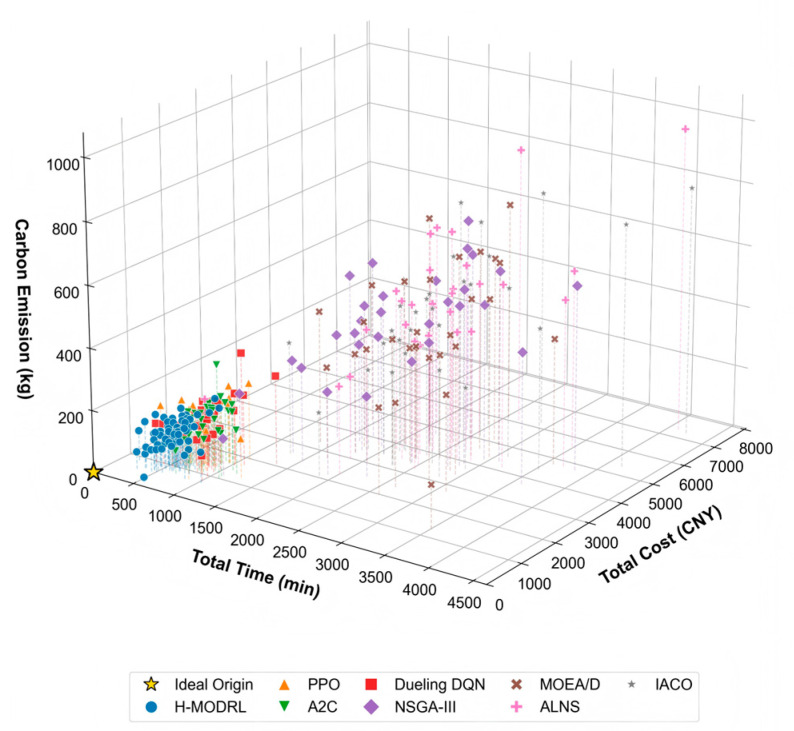
3D Pareto front spatial distribution—Scenario 2.

To evaluate robustness under a high-dimensional, sparse, real-world road network (Scenario 2), we benchmark H-MODRL against seven baselines from the DRL group (A2C, Dueling DQN, Vanilla PPO) and the metaheuristic group (NSGA-III, MOEA/D, ALNS, IACO). Compared to Scenario 1, Scenario 2 substantially increases the problem scale and topological complexity, thereby imposing stringent demands on generalization and search efficiency. The Pareto front visualizations (Figure 6 and Figure 7) reveal a distinct separation among the competing methods. Specifically, H-MODRL maintains a tightly clustered non-dominated set near the ideal point, demonstrating sustained dominance even within a non-convex objective space. While the DRL baselines remain somewhat competitive, their solutions predominantly lie on the outer envelope of the H-MODRL front and are strictly dominated within the cost–time projection, with most solutions concentrated in the 1300–1500 CNY cost band. Crucially, the heuristic baselines degrade sharply, yielding sparse solutions distributed across a distinctly inferior region (cost > 3500 CNY; time > 2000 min), which is consistent with entrapment in sub-optimal local basins under heightened terrain-induced constraints.

**Figure 8:**
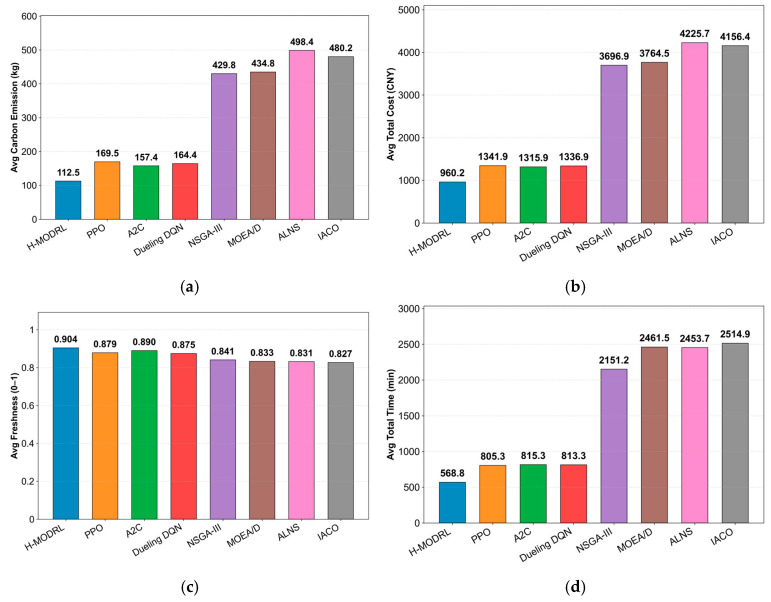
Comparison of multi-objective performance in Scenario 2. (**a**) Average total cost; (**b**) Average carbon emissions; (**c**) Average freshness; (**d**) Average total time.

**Figure 9:**
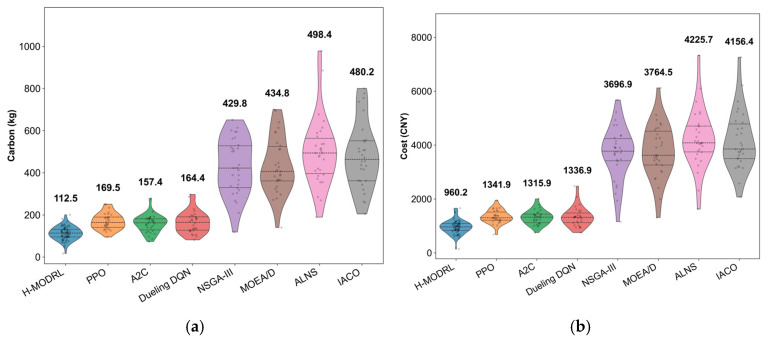

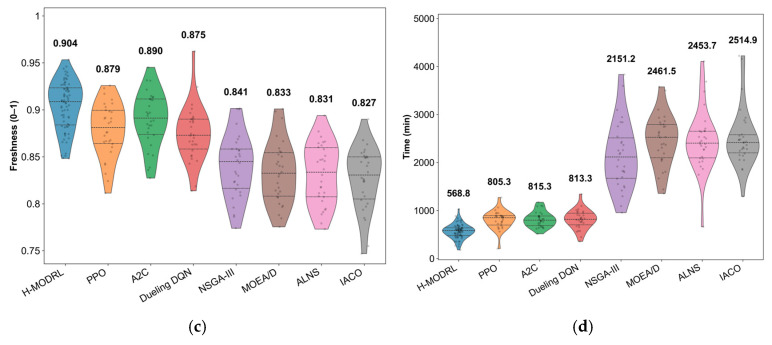
Distribution of performance indicators in Scenario 2. (**a**) Cost; (**b**) Carbon emissions; (**c**) Freshness; (**d**) Time.

This structural advantage translates into consistent gains across all operational objectives (Figure 8a–d). Specifically, H-MODRL achieves the optimal mean performance, yielding an average total cost of 960.2 CNY, total carbon emissions of 112.5 kg, an average terminal freshness level of 0.907, and a total delivery time of 568.8 min. Relative to Vanilla PPO, H-MODRL reduces total cost and emissions by 28.4% and 33.6%, respectively, improves time efficiency by 29.4%, and preserves a 3.5% freshness advantage. This indicates that the benefits of the hybrid-evolution mechanism and physics-based guidance amplify as network complexity increases. Furthermore, compared to the worst-performing heuristics, the performance margins are substantially larger (e.g., a 77.3% cost reduction and 77.4% emission reduction versus ALNS, and a 77.4% delivery time reduction versus IACO), alongside a 17.5–19.0% freshness gain. This demonstrates the strong resilience of the proposed method under extreme operating conditions. The violin plots (Figure 9a–d) further substantiate this distributional robustness. Across all metrics, H-MODRL exhibits highly compact, low-variance distributions with an absence of pronounced heavy tails. In contrast, the RL baselines show broader spreads, and the heuristic group displays severe heavy-tailed behavior with extreme outliers (e.g., emissions > 1000 kg; time > 4000 min; freshness occasionally dropping below 0.4). These findings highlight the substantial instability of traditional baselines when navigating sparse, rugged networks.

### 4.4. Performance Evaluation in Mountainous Terrain

Scenario 3 represents the largest and most challenging setting, characterized by long-haul deliveries within a rugged mountainous network. This environment places stringent demands on global planning, constraint satisfaction, and stability under stochasticity. We evaluate H-MODRL against seven baselines from the DRL group (A2C, Dueling DQN, Vanilla PPO) and the metaheuristic group (NSGA-III, MOEA/D, ALNS, IACO) based on Pareto-front topology, mean physical metrics, and distributional robustness.

**Figure 10:**
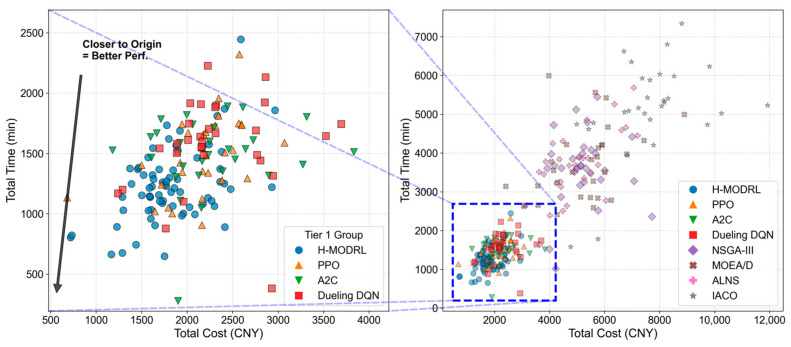
Pareto solution distribution—Scenario 3.

**Figure 11:**
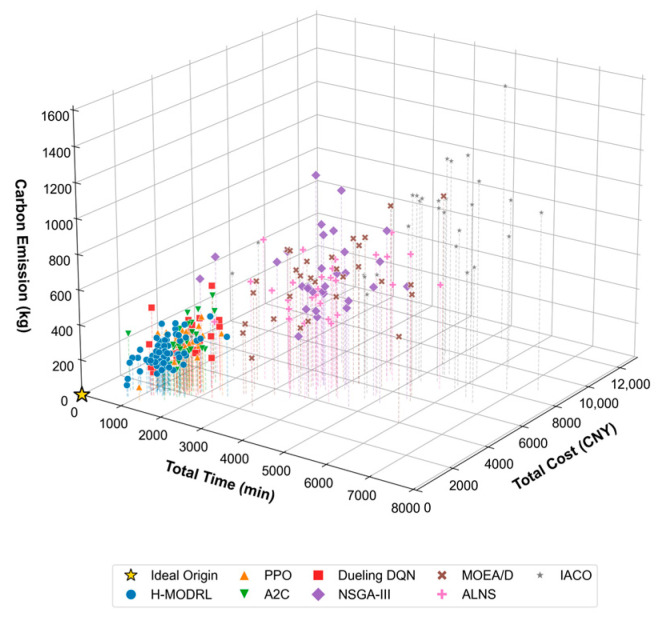
3D Pareto front spatial distribution—Scenario 3.

The Pareto front visualizations (Figure 10 and Figure 11) demonstrate that H-MODRL preserves clear dominance even within this extreme regime. Its non-dominated solutions concentrate in the best-known region (approximately 1800 CNY in cost and 1200 min in delivery time) and form a compact, near-spherical cluster in the 3D objective space. This indicates strong convergence toward the ideal point and a stable trade-off among cost, time, and carbon emissions while successfully preserving commodity freshness. Although the DRL baselines remain the closest competitors, their fronts largely envelop, rather than reach, the H-MODRL boundary. In the cost–time projection, they are concentrated around 2000–2500 CNY and are strictly dominated, suggesting a limited ability to consistently attain the optimal frontier under high constraint pressure. In contrast, the heuristic baselines degrade severely, producing sparse solutions distributed across clearly inferior regions (cost > 5000 CNY; time > 3500 min). This behavior is consistent with entrapment in sub-optimal local basins as network scale and sparsity increase.

**Figure 12:**
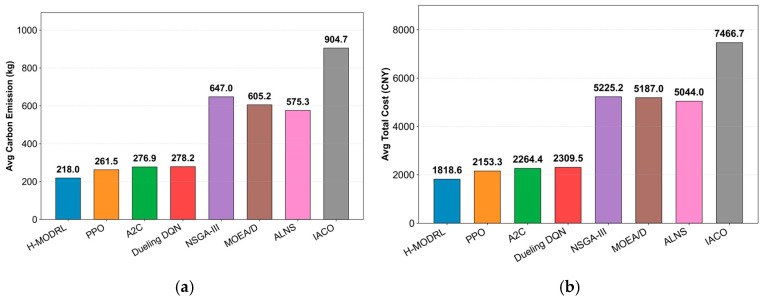

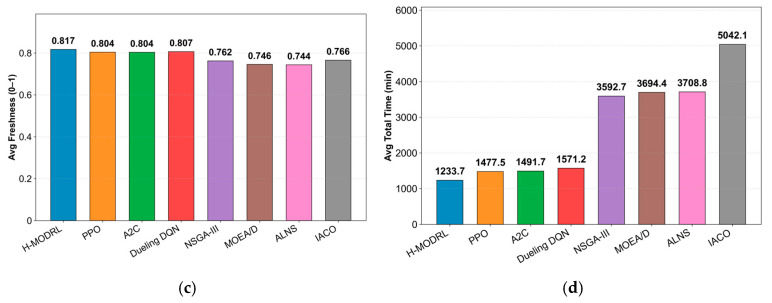
Comparison of multi-objective performance in Scenario 3. (**a**) Average total cost; (**b**) Average carbon emissions; (**c**) Average freshness; (**d**) Average total time.

**Figure 13:**
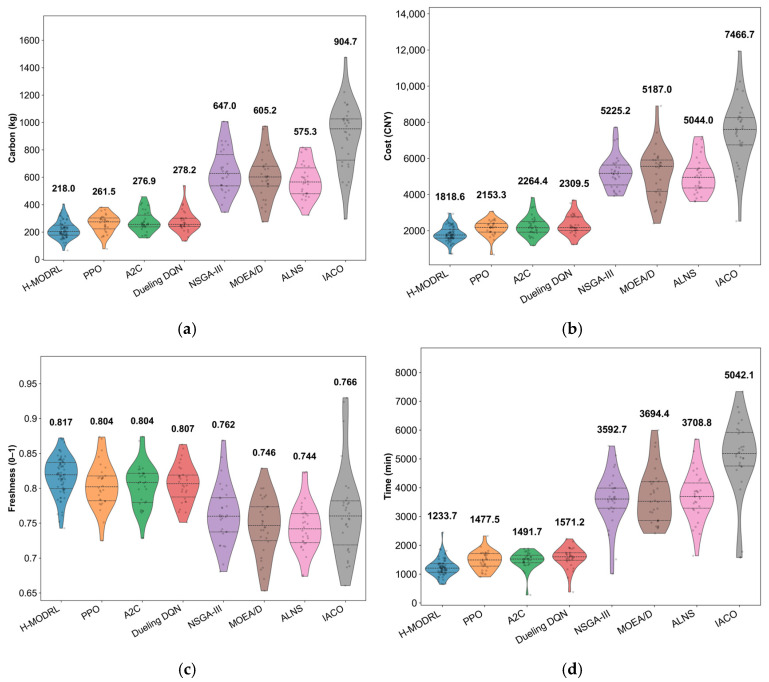
Distribution of performance indicators in Scenario 3. (**a**) Cost; (**b**) Carbon emissions; (**c**) Freshness; (**d**) Time.

These structural differences translate into consistent operational gains across all metrics (Figure 12a–d). Specifically, H-MODRL achieves the optimal mean performance, yielding an average total cost of 1818.6 CNY, total carbon emissions of 218.0 kg, and a total delivery time of 1233.7 min. Simultaneously, it delivers the highest average terminal freshness (0.814, tying with A2C and performing marginally above PPO at 0.811). Relative to Vanilla PPO, H-MODRL reduces total cost, emissions, and delivery time by 15.5%, 16.6%, and 16.5%, respectively, while retaining a marginal yet consistent freshness advantage. Furthermore, the performance margins against the weakest heuristic baseline are substantially larger (e.g., a 75.6% cost reduction and 75.9% emission reduction versus IACO), alongside a 13.8–14.7% improvement in freshness over traditional methods. This indicates markedly better protection against long-distance freshness degradation. The violin plots (Figure 13a–d) further substantiate this distributional robustness. Across all metrics, H-MODRL exhibits highly compact, low-variance distributions with minimal tail risk. In contrast, the DRL baselines display wider spreads, and the heuristic group demonstrates severe heavy-tailed behavior with extreme outliers (e.g., emissions exceeding 1500 kg, delivery time approaching 9000 min, and freshness collapsing to 0.2–0.4). These findings highlight the poor constraint compliance and inherent instability of traditional baselines when navigating large, sparse mountainous networks.

### 4.5. Hyperparameter Optimization and Convergence Dynamics

**Figure 14:**
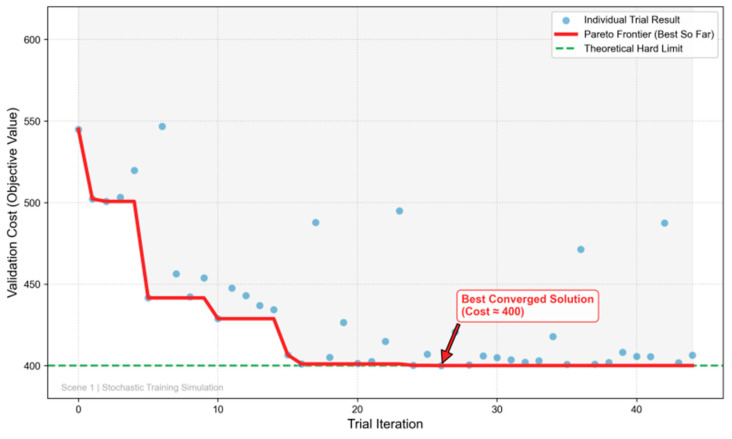
Scenario 1—Hyperparameter optimization.

**Figure 15:**
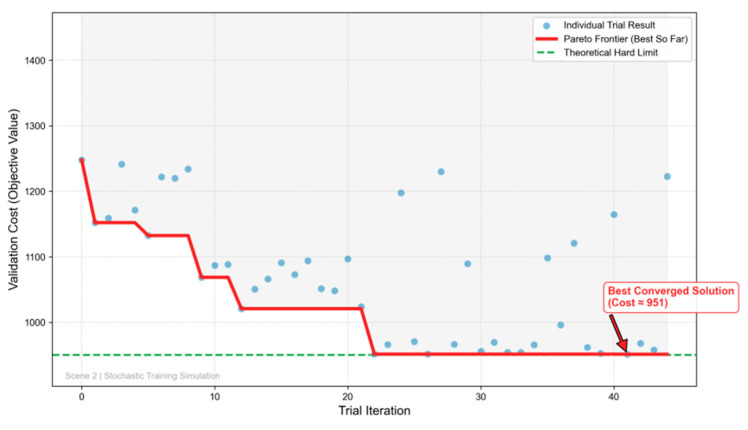
Scenario 2—Hyperparameter optimization.

**Figure 16:**
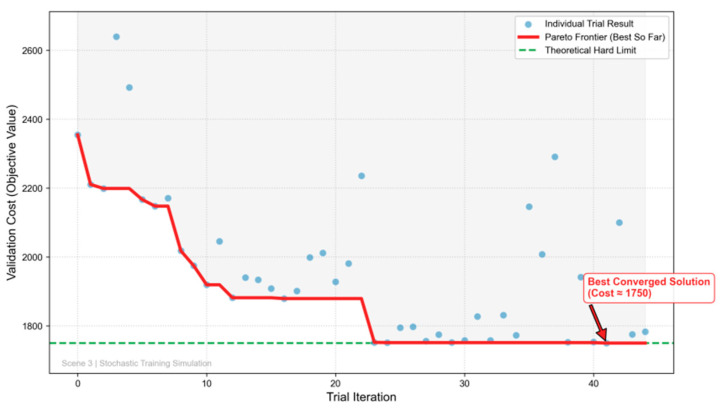
Scenario 3—Hyperparameter optimization.

To maximize the cross-scenario generalization of H-MODRL, we apply Bayesian hyperparameter optimization using Optuna with a Tree-structured Parzen Estimator (TPE). Key hyperparameters—including the learning rate, discount factor, and entropy coefficient—are automatically searched over 45 trials. Figure 14, Figure 15 and Figure 16 summarize the evolution of the validation cost across the three geographic scenarios. In Scenario 1 (plain terrain), the optimization exhibits rapid and stable convergence. The validation cost drops steeply within the first few trials (approximately Trials 0–5) and subsequently reaches a near-optimal plateau. The best configuration is identified at Trial 26, after which the Pareto frontier remains essentially flat despite occasional high-variance exploratory trials. In Scenario 2 (hilly terrain), the search trajectory becomes stepwise, reflecting a highly non-convex landscape. Early trials yield incremental improvements, followed by a pronounced “phase transition” around Trials 20–22 that shifts the search into a superior hyperparameter region; the global optimum is only stabilized later, around Trial 41. Finally, in Scenario 3 (mountainous terrain), convergence is robust yet more gradual. The cost compresses rapidly during the first 10 trials, followed by sustained marginal gains characterized by a jagged, progressive decline. The optimal configuration is again confirmed near Trial 41, a behavior consistent with the heightened rigidity imposed by long-haul constraints.

A cross-scenario comparison reveals three consistent patterns. First, convergence time increases with terrain complexity. The plain scenario converges relatively early (Trial 26), whereas both the hilly and mountainous scenarios demand an extended search budget (Trial 41). This implies that practical deployments in complex road networks must allocate larger computational budgets to prevent under-tuning. Second, even after aggressive TPE optimization, the best achievable validation costs exhibit a clear hierarchy: Scenario 1 ≈400 < Scenario 2 ≈951 < Scenario 3 ≈1750. This strict ordering quantifies the hard lower bounds imposed by geographic and physical constraints. Third, across all scenarios, although individual trials exhibit high variance, the convergence curve of the Pareto frontier remains monotonically non-increasing. This behavior substantiates the robustness of TPE-based Bayesian optimization within high-dimensional, non-convex hyperparameter spaces.

### 4.6. Ablation Study: Quantitative Analysis of H-MODRL Core Modules

**Figure 17:**
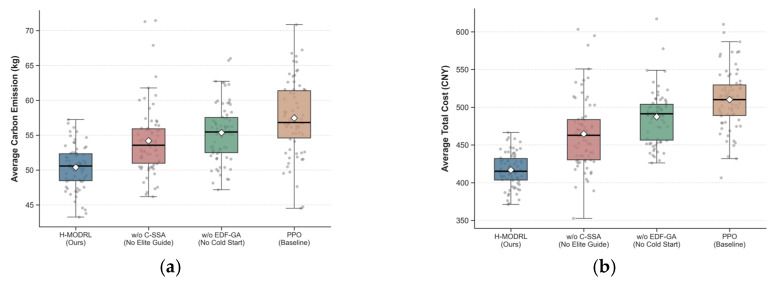

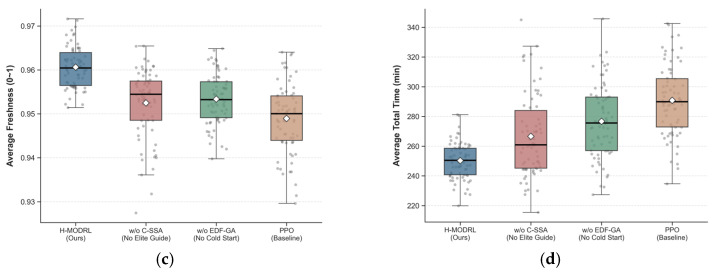
Ablation study results—Scenario 1 (Plain). Box-and-scatter combination plots comparing PPO (Baseline), w/o EDF-GA, w/o C-SSA, and H-MODRL (Ours) across four metrics: (**a**) Average total cost; (**b**) Average total time; (**c**) Average carbon emissions; (**d**) Average freshness.

**Figure 18:**
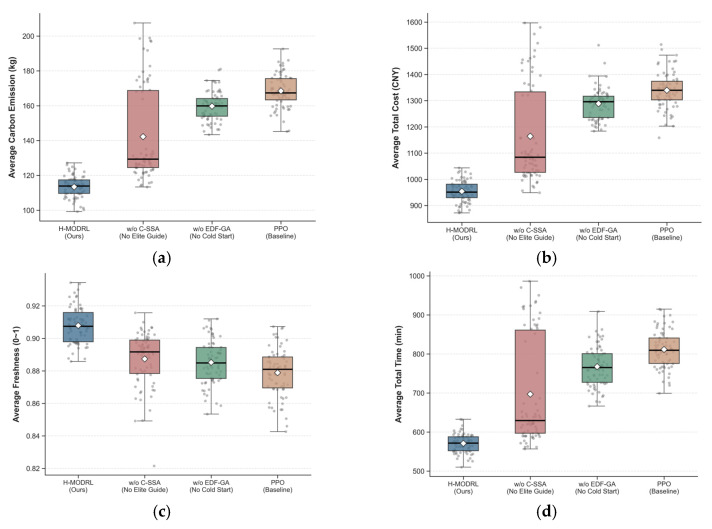
Ablation study results—Scenario 2 (Hilly). Box-and-scatter combination plots comparing PPO (Baseline), w/o EDF-GA, w/o C-SSA, and H-MODRL (Ours) across four metrics: (**a**) Average total cost; (**b**) Average total time; (**c**) Average carbon emissions; (**d**) Average freshness.

**Figure 19:**
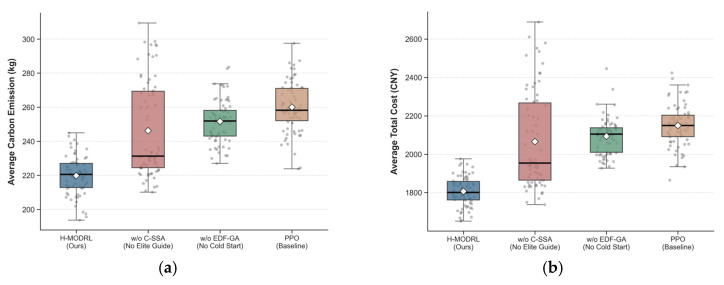

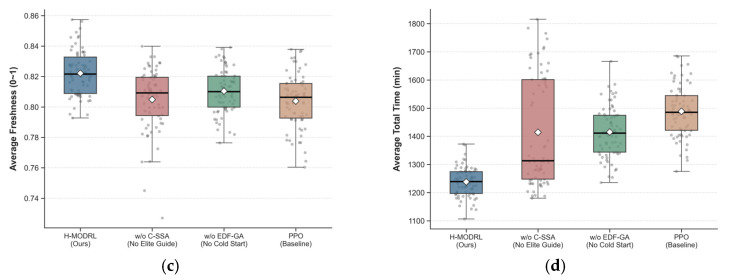
Ablation study results—Scenario 3 (Mountainous). Box-and-scatter combination plots comparing PPO (Baseline), w/o EDF-GA, w/o C-SSA, and H-MODRL (Ours) across four metrics: (**a**) Average total cost; (**b**) Average total time; (**c**) Average carbon emissions; (**d**) Average freshness.

To rigorously investigate the independent marginal contribution of each innovative module within the proposed H-MODRL algorithm, this section takes Vanilla PPO (a pure deep reinforcement learning network) as the control baseline and constructs two ablation variants by respectively removing the Earliest Deadline First Genetic Algorithm (EDF-GA) cold-start mechanism (denoted as w/o EDF-GA) and the Chaotic Sparrow Search Algorithm (C-SSA) elite guidance strategy (denoted as w/o C-SSA). Experiments were independently repeated 70 times across the three heterogeneous simulation scenarios—plain (Scenario 1), hilly (Scenario 2), and mountainous (Scenario 3). The statistical means of the four core physical indicators for each ablation variant are summarized in Table 3, and the full per-scenario comparison is visualized in Figure 17, Figure 18 and Figure 19 using box-and-scatter combination plots that simultaneously display the median, interquartile range, full distribution shape, and individual data points.

#### 4.6.1. Effectiveness of the EDF-GA Cold-Start Mechanism: Initial State-Space Dimensionality Reduction

Removing the EDF-GA module (w/o EDF-GA) leads to rigid performance degradation across all three terrain types, with severity escalating as terrain complexity increases, as shown in Table 3 and Figure 17, Figure 18 and Figure 19. In the plain scenario, the absence of EDF-GA causes the average total cost to rise from 420.3 CNY to 482.5 CNY (+14.8%) and the average total time to extend from 249.2 min to 278.0 min (+11.6%). The degradation amplifies substantially in the hilly scenario, where w/o EDF-GA incurs a cost increase from 960.2 CNY to 1280.5 CNY (+33.4%) and a time increase from 568.8 min to 770.0 min (+35.4%). Under the extreme mountainous conditions, the average total cost escalates from 1818.6 CNY to 2080.5 CNY (+14.4%), with the average delivery time increasing by approximately 186.3 min (+15.1%). The carbon emissions metric exhibits a parallel degradation trend across all three scenarios: +11.2% (Scenario 1), +42.2% (Scenario 2), and +15.6% (Scenario 3). The freshness metric shows relatively smaller but consistent degradation, with the baseline PPO already achieving competitive freshness values due to the physics-based reward guidance.

Mechanism analysis: Deep Reinforcement Learning (DRL) models, when confronted with cold chain VRP scenarios involving hard time windows and complex physical constraints, suffer from a severe sparse-reward dilemma. Upon removal of EDF-GA, the PPO network is forced to commence exploration from a completely random, low-quality state space, expending substantial computational resources on trial-and-error attempts at infeasible solutions. Although the subsequent C-SSA stage continues to assist in optimization, the disadvantageous “starting line” of the algorithm causes its final convergence lower bound to be permanently locked in an inferior region. The wider interquartile ranges and extended whiskers of the w/o EDF-GA distributions in the box-and-scatter plots confirm that cold-start deficiency not only degrades mean performance but also inflates solution variance, indicating reduced algorithmic stability. These results quantitatively demonstrate the irreplaceable role of the EDF-GA mechanism in providing a high-quality initial population and achieving state-space dimensionality reduction during the early convergence phase.

#### 4.6.2. Effectiveness of the C-SSA Elite Guidance Mechanism: Escaping Local Optima

The w/o C-SSA variant reveals a pronounced terrain-dependent degradation pattern, as evidenced by the distributional shifts visible in Figure 17, Figure 18 and Figure 19. In the low-complexity plain scenario, removing the elite guidance mechanism results in relatively manageable performance loss: the average total cost rises from 420.3 CNY to 466.0 CNY (+10.9%), and the average total time increases from 249.2 min to 270.0 min (+8.3%). However, the deterioration becomes critically pronounced in the high-dimensional, non-convex solution spaces of the hilly and mountainous scenarios. In Scenario 2, w/o C-SSA yields an average total cost of 1170.0 CNY—a 21.8% increase over the complete H-MODRL—and the average total time extends to 704.0 min (+23.8%). In Scenario 3, the comprehensive performance of w/o C-SSA approaches and, on the carbon emissions metric (245.0 kg vs. 252.0 kg for w/o EDF-GA), partially surpasses the degradation caused by removing the cold-start mechanism, indicating that the elite guidance function becomes even more structurally critical than the initialization stage under extreme terrain constraints.

Mechanism analysis: The mean values of w/o C-SSA are forcibly elevated by a large number of extreme inferior solutions exhibiting a pronounced “heavy-tail effect”—a pattern that is visually evident in the elongated upper whiskers and scattered outlier points of the w/o C-SSA box-and-scatter distributions, particularly in Scenario 2 and Scenario 3. Within the sparse mountainous road network, the PPO network is highly susceptible to “policy collapse” during the mid-to-late training phase, becoming trapped in dead-end loops of suboptimal routes. The removal of the C-SSA mechanism deprives the algorithm of both the “sentinel random escape” capability and the expert-guided search capacity. The progressively widening performance gap between w/o C-SSA and H-MODRL across the scenario gradient (Scenarios 1 → 2 → 3) quantitatively confirms that swarm intelligence-based elite guidance is indispensable for maintaining robust optimization in increasingly rugged, high-dimensional solution landscapes.

#### 4.6.3. Cross-Scenario Compound Gains and Comprehensive Baseline Superiority

The complete H-MODRL algorithm achieves comprehensive performance dominance across all three scenarios, as evidenced by the consistent leftmost (most favorable) positioning of its distributions across all four metric panels. Compared with the baseline Vanilla PPO, H-MODRL reduces the average total cost by 17.8%, 28.4%, and 15.5% in the plain, hilly, and mountainous scenarios, respectively. The carbon emission reductions follow a parallel trajectory: 13.9% (Scenario 1), 33.6% (Scenario 2), and 16.6% (Scenario 3). The freshness metric, while exhibiting the narrowest absolute gaps due to the inherently bounded [0, 1] scale, consistently favors H-MODRL with improvements of 1.1%, 2.8%, and 1.6% over the Vanilla PPO baseline across the three scenarios.

Notably, the increase in terrain complexity produces a significant “penalty amplification” effect. In Scenario 3, the pure PPO algorithm, lacking the protection of heuristic rules, frequently triggers time-window violations and long-distance detours, yielding not only elevated mean values but also substantially wider distributions across all four metrics. In contrast, through the dual empowerment of Stage 1 (GA delineating a high-quality feasible region) and Stage 2 (SSA escorting to prevent premature convergence), H-MODRL successfully compensates for the inherent deficiencies of pure DRL algorithms under rigid physical constraints. The compact box-and-scatter distributions of H-MODRL across all scenarios and metrics—characterized by narrow interquartile ranges, short whiskers, and minimal outlier points—fully substantiate the outstanding engineering reliability of this physics-aware hybrid architecture in practical agricultural cold chain logistics deployment.

#### 4.6.4. Limitations of the Ablation Design

We note a limitation of the current ablation design: the physics-based reward function and the elite guiding term are not fully isolated as independent ablation factors. The complete H-MODRL framework contains three tightly coupled stages, and the MO-PPO decision stage internally integrates two interdependent learning signals: (i) the physics-based compound reward R_physics, which scalarizes the four objectives (cost, time, carbon, freshness) with weights w1-w4 and embeds the Arrhenius-based freshness-decay term Delta_Q_loss; and (ii) the elite guidance term I(a_t in G), which provides positive reinforcement when the agent’s actions match route fragments from the C-SSA-constructed Elite Guiding Set. These two signals operate synergistically during PPO training—the physics-based reward defines the optimization landscape, while the elite guidance term constrains the effective exploration space toward high-quality Pareto regions. Completely removing the physics-based reward would fundamentally alter the optimization landscape (the learning problem would no longer correspond to the original four-objective ACCL-RP formulation), rendering fair comparison with the complete H-MODRL infeasible under the same experimental conditions. Conversely, removing only the elite guidance term while retaining all other components would isolate its contribution, but this variant is not equivalent to the w/o C-SSA condition tested here, because w/o C-SSA removes the entire Stage 2 (including chaotic initialization, LNS-enhanced search, and the elite set construction), not merely the guidance term within MO-PPO. Due to these inherent coupling constraints, the current ablation design removes entire algorithmic stages (w/o EDF-GA and w/o C-SSA) rather than their internal sub-components. While this stage-level ablation convincingly demonstrates that each major module (GA-EDF cold-start initialization and C-SSA-LNS elite guidance) contributes essentially and non-redundantly to overall performance—with removal of either stage causing consistent degradation across all three terrain scenarios and all four metrics as shown in Table 3 and Figure 17, Figure 18 and Figure 19—it cannot attribute performance gains to specific sub-mechanisms within each stage. For instance, the performance improvement of the complete H-MODRL over w/o C-SSA cannot be decomposed into the separate contributions of chaotic initialization, the LNS destroy-repair operator, and the elite imitation guidance term. Future work should investigate more granular ablation designs, such as: (a) replacing the physics-based reward with a standard uniformly-weighted scalarization while keeping the elite guidance term fixed; (b) varying the elite guidance coefficient lambda_guide in isolation (e.g., lambda_guide in {0, 0.25, 0.5, 0.75, 1.0}) to quantify its marginal effect; and (c) testing C-SSA variants with and without the LNS operator to isolate the contribution of the neighborhood search enhancement. These finer-grained analyses would further disentangle the individual contributions of the coupled sub-components within each stage.

## 5. Conclusions

To address the challenges of Agricultural Cold Chain Logistics (ACCL)—where operational objectives are sharply conflicting and tightly coupled to terrain-dependent energy consumption under frequent disruptions—we propose and validate a physics-based Hybrid Multi-Objective Deep Reinforcement Learning (H-MODRL) framework. This framework integrates a hierarchical coupling mechanism comprising a heuristic warm start, evolutionary policy guidance, and deep reinforcement learning-based decision-making. This integration enables stable, Pareto-efficient routing that jointly optimizes logistics cost, delivery time, carbon emissions, and terminal freshness across heterogeneous geographic regions. Across Scenarios 1–3, H-MODRL consistently anchors the non-dominated set near the ideal point in both 2D and 3D Pareto visualizations, forming compact clusters rather than the scattered, diverging fronts typical of conventional metaheuristics (e.g., MOEA/D, ALNS). This performance indicates that prior-guided hybridization effectively mitigates sparse-reward exploration and cold-start failures inherent to non-convex, high-dimensional spatiotemporal routing. Consistent with these findings, the TPE-based Bayesian optimization via Optuna converges within 26–41 trials, with delayed convergence observed in more complex terrains. This confirms that the hybrid design enhances learnability and stabilizes optimization under increasing topological difficulty. Furthermore, the systematic ablation experiments reported in Section 4.6 quantitatively confirm that the EDF-GA cold-start mechanism and the C-SSA elite guidance strategy each deliver essential, non-redundant marginal contributions to overall algorithmic performance, with their respective removal leading to rigid performance degradation across all terrain types.

Ultimately, H-MODRL establishes a new state-of-the-art baseline for trade-offs among cost efficiency, carbon reduction, freshness preservation, and systemic robustness. In the most demanding mountainous setting (Scenario 3), the proposed method achieves an average total cost of 1818.6 CNY and carbon emissions of 218.0 kg. These results correspond to drastic reductions of 75.6% and 75.9%, respectively, relative to the IACO baseline (7466.7 CNY and 904.7 kg). Simultaneously, H-MODRL maintains the highest average terminal freshness (0.814) and the shortest mean delivery time (1233.7 min), representing a 75.5% reduction in time compared to the worst-performing baseline (5042.1 min). Furthermore, distributional analyses demonstrate that H-MODRL yields compact, low-variance performance profiles without pronounced heavy tails or outliers. In contrast, heuristic baselines exhibit heavy-tailed instability and occasional drastic freshness degradation, while DRL baselines display wider performance spreads under extreme conditions. Collectively, these results substantiate the engineering reliability and practical viability of the proposed perception-to-decision re-optimization framework for uncertain ACCL environments.

Despite the strong performance of H-MODRL in single-vehicle and mid-scale fleet scheduling, this study presents several limitations that warrant consideration when interpreting the results:

1. Sensitivity to reward weights and incomplete ablation isolation: The current study employs a fixed default weight configuration (w1 = 0.35, w2 = 0.15, w3 = 0.25, w4 = 0.25) for the scalarized compound reward. However, weight selection critically determines the Pareto trade-off behavior in scalarized multi-objective RL, and different application scenarios (e.g., cost-sensitive versus freshness-prioritized dispatching) may necessitate distinct weight configurations. Furthermore, as acknowledged in Section 4.6.4, the physics-based reward function and the elite guidance term are not fully isolated as independent ablation factors—the current ablation design removes entire algorithmic stages (w/o EDF-GA, w/o C-SSA) rather than their internal sub-components, and therefore cannot attribute performance gains to specific sub-mechanisms (e.g., the Arrhenius reward term versus the elite imitation term) within each stage. Future research should (i) systematically investigate the impact of weight variations on individual objective attainment through global sensitivity analysis, and (ii) conduct more granular ablation studies (e.g., varying the elite guidance coefficient lambda_guide in isolation, or testing C-SSA variants with and without the LNS operator) to disentangle the respective contributions of the coupled sub-components.
2. Scalability to massive fleets: Scaling the framework to accommodate very large fleets may present challenges for centralized training paradigms due to exponential state-space growth and communication bottlenecks. Future work will explore distributed Multi-Agent Reinforcement Learning (MARL), potentially leveraging mean-field formulations or attention-based communication architectures, to enable decentralized coordination amid congestion interactions.
3. Statistical significance verification: The current empirical analysis relies on mean comparisons across 70 (H-MODRL) and 30 (baseline) independent runs, full distributional visualization via violin plots, and multi-objective Pareto-front dominance analysis, without formal statistical hypothesis testing (e.g., the Wilcoxon rank-sum test). We acknowledge this as a limitation and offer the following clarification. The evaluation employs a single custom-constructed dataset whose three scenarios form a deliberate difficulty gradient (plain → hilly → mountainous) rather than independent, identically distributed problem instances from a common population, which is the standard prerequisite for valid cross-scenario rank-based testing. To the best of our knowledge, no publicly available benchmark suite exists for agricultural cold chain logistics that jointly models Arrhenius-based freshness decay, full life-cycle carbon emissions, hard time windows, and dynamic disruptions at the county level, making cross-dataset validation currently infeasible. As detailed in Section 4.1, we instead provide a multi-faceted evidence base: (i) 70/30 independent runs per scenario yield stable estimates; (ii) violin plots disclose full empirical distributions, with H-MODRL consistently exhibiting the narrowest interquartile ranges and minimal tail risk; (iii) 2D/3D Pareto-front visualizations confirm that H-MODRL’s non-dominated set strictly dominates the objective regions of all competing methods, which is a stronger and more interpretable comparison criterion than scalar hypothesis tests in multi-objective optimization; and (iv) H-MODRL achieves the best mean on all four objectives across all three scenarios, with improvements of 13.4–33.6% over the strongest baseline. Given the unique nature of the dataset and the consistency of the evidence across qualitatively distinct terrain conditions, the addition of formal hypothesis testing is unlikely to alter the conclusions. Future work will incorporate formal statistical procedures should suitable multi-instance benchmarks become available.
4. Generalization capability: The proposed model has been validated primarily across three representative Chinese county-level scenarios. Its generalization performance across disparate road network topologies and the cold chain operational norms of different countries and regions remains to be empirically verified.
5. Reproducibility: The experiments in this study employ a custom-constructed heterogeneous terrain simulation dataset built from multiple third-party data sources, including: (i) rural vector road network and geographic feature data from the National Platform for Common Geospatial Information Services (Tianditu) and the National Geographic Information Resources Directory Service System; (ii) 30 m-resolution DEM terrain data from the Resource and Environment Science and Data Center, Chinese Academy of Sciences; (iii) long-series meteorological measurement data from the National Meteorological Science Data Center, China Meteorological Administration; (iv) historical rural road traffic condition data from Gaode Map (Amap) and Baidu Map open platforms; and (v) de-identified cold chain enterprise operational data from the Alibaba Tianchi and HeyWhale (Kesci) public datasets. These raw third-party data are subject to their respective usage terms and licensing agreements and cannot be redistributed as part of this publication. Furthermore, to the best of our knowledge, no publicly available standardized benchmark currently exists for agricultural cold chain logistics that jointly integrates Arrhenius-based freshness-decay kinetics, full life-cycle carbon emission modeling, hard time-window constraints, and stochastic dynamic disruption modeling at the county operational scale. The complete parameter configurations of the three simulation scenarios are documented in Table 1 and Table 2, and the disruption-generation rules are mathematically specified in Section 2.1 (Equations for delta_k and travel time under disruption) and Section 4.1 (Table 1 and the detailed parameter descriptions). Additional implementation details and environment configuration files may be obtained from the corresponding author upon reasonable request, subject to the third-party data usage restrictions noted above. The development of a publicly available, standardized multi-instance benchmark for agricultural cold chain logistics optimization is identified as an important direction for community-wide collaborative effort.

Furthermore, future work will focus on developing an edge-cloud digital twin pipeline. This initiative aims to deploy lightweight inference models on vehicle-side edge devices while continuously assimilating real-time road condition streams, thereby extending the optimization scope from strategic routing to real-time, vehicle-dynamics-aware control.

## Figures and Tables

**Table 1:** Parameter settings for simulation scenarios.

Scene Type	Geographic Area (km^2^)	Nodes N	Road-Network Topology	Disturbance Rate λ_dist_
Scene-1 (Plain)	50	84	Regular grid, high density 2.1 km/km^2^	Low
Scene-2 (Hilly)	120	68	Random layout, medium density 0.95 km/km^2^	Medium
Scene-3 (Mountainous)	280	50	Sparse tree-like, low density 0.45 km/km^2^	High

**Table 2:** Core hyperparameter configuration of H-MODRL.

Module	Parameter	Value
Stage I (GA)	Population size N_pop_/iterations G_max_	100/200
Stage II (C-SSA)	Producer ratio R_prod_/scout ratio R_scout_	0.2/0.1
Stage III (PPO)	Learning rate η_lr_	1 × 10^−5^~1 × 10^−2^
	Discount factor γ/clip threshold ε	0.90~0.999/0.1~0.4
	Guidance coefficient λ_guide_	0.5 (linear decay)

**Table 3:** Quantitative comparison of core module ablation results across three heterogeneous scenarios (mean values).

Scenario	Metric	PPO (Baseline)	w/o EDF-GA	w/o C-SSA	H-MODRL (Ours)
Scenario 1 (Plain, low complexity)	Avg. Total Cost (CNY)	511.4	482.5	466.0	420.3
	Avg. Total Time (min)	287.8	278.0	270.0	249.2
	Avg. Carbon Emissions (kg)	58.0	55.5	54.0	49.9
	Avg. Freshness (0~1)	0.949	0.952	0.951	0.959
Scenario 2 (Hilly, medium complexity)	Avg. Total Cost (CNY)	1341.9	1280.5	1170.0	960.2
	Avg. Total Time (min)	805.3	770.0	704.0	568.8
	Avg. Carbon Emissions (kg)	169.5	160.0	141.5	112.5
	Avg. Freshness (0~1)	0.879	0.882	0.886	0.904
Scenario 3 (Mountainous, high complexity)	Avg. Total Cost (CNY)	2153.3	2080.5	2074.0	1818.6
	Avg. Total Time (min)	1477.5	1420.0	1428.0	1233.7
	Avg. Carbon Emissions (kg)	261.5	252.0	245.0	218.0
	Avg. Freshness (0~1)	0.804	0.807	0.803	0.817

## Data Availability

The data supporting the findings of this study are available from the corresponding author upon reasonable request.
